# Supplementary material for: Hierarchical progressive learning of cell identities in single-cell data
Source: Nat Commun. 2021 May 14;12:2799. doi: 10.1038/s41467-021-23196-8 (PMC8121839; doi:10.1038/s41467-021-23196-8)
Supplement: Supplementary file 1 — Supplementary Information [file 41467_2021_23196_MOESM1_ESM.pdf]

Supplementary data for:

Hierarchical progressive learning of cell identities in single-cell data

Lieke Michielsen[1,2,3] (l.c.m.michielsen@tudelft.nl)
Marcel J.T. Reinders[1,2,3] (m.j.t.reinders@tudelft.nl)
Ahmed Mahfouz[1,2,3]* (a.mahfouz@lumc.nl)

[1] Department of Human Genetics, Leiden University Medical Center, Einthovenweg 20, 2333ZC, Leiden, The Netherlands
[2] Leiden Computational Biology Center, Leiden University Medical Center, Einthovenweg 20, 2333ZC, Leiden, The Netherlands
[3] Delft Bioinformatics Lab, Delft University of Technology, Van Mourik Broekmanweg 6, 2628XE, Delft, The Netherlands

* Corresponding author (a.mahfouz@lumc.nl)

Supplementary Note 1

When matching the cell populations from two datasets, we distinguish five options: simple, multiple columns, multiple rows, complex, and impossible. When describing the different scenarios within these options, we sometimes make a distinction between leaf nodes and internal nodes. Here, it is important to remember that only *T1* can have internal nodes since this is the tree that is updated. *T2* is always a flat classification tree, so only consists of the root node and leaf nodes.

Simple

In this scenario, we find a unique match between a cell population, P_i, from dataset 1 and a cell population, P_j, from dataset 2. As as consequence, $X_{j,I}$ will be 1 or 2 and the rest of row *j* and column *i* in *X* are zero. Within this scenario, there are three different options:

1. Both cell populations are leaf or internal nodes. This indicates a perfect match. The tree is not updated, but the labels of P_j are renamed to P_i (Supplementary Fig. 24A). This is the same scenario as the 'perfect match' scenario described in the main text.
2. P_i is a leaf or internal node, but P_j is the root node of *T2*. This indicates that P_i is missing in dataset 2. The node, however, is already in the tree, so it is not updated (Supplementary Fig. 24B).
3. P_i is the root of *T1*, but the P_j is a leaf node. This indicates that P_j is missing in dataset 1. The cell population is thus also not in the tree yet, so we will add it as a child to the root (Supplementary Fig. 24C). This is the same scenario as the 'new population' scenario described in the main text.

Multiple rows

In this scenario, a cell population, P_i, from dataset 1 matches multiple populations from dataset 2. In *X* there will be multiple non-zero values in column *i.* Here, we distinguish two different scenarios:

1. P_i matches only cell populations from dataset 2 that are leaf node. We consider the cell populations from dataset 2 subpopulations of P_i, so we add them as descendants to P_i (Supplementary Fig. 25A). This is the same scenario as the 'splitting nodes' scenario described in the main text.
2. The root node of *T2* is also involved. We simple ignore this node and for the rest do the same as above (Supplementary Fig. 25B-C).

Multiple columns

This scenario is quite similar to the multiple rows scenario. Here, however, multiples populations from dataset 1 match one cell population, P_j, of dataset 2. In *X* there will be multiple non-zero values in row *j.* This scenario is a little more complex since the populations from dataset 1 does not have to be leaf nodes or the root node, but there can also be internal nodes in this tree. Here, we distinguish three different scenarios:

1. The root node of *T1* and *T2* are not involved, so multiple cell populations, which can be leaf or internal nodes, from dataset 1 match P_j. We consider the cell populations from dataset 1 subpopulations of P_j, so we need to add P_j as a parent node to these cell populations. (Supplementary Fig. 26A). This is same scenario as the 'merging nodes' scenario described in the main text. It could be, however, that this node already exists in this tree. (Supplementary Fig. 26B). If this is the case, we have a perfect match between a node from tree 1 and tree 2, so we do not have to update the tree, but we only have to update the labels of P_j.

2. Besides leaf or internal nodes, the root of *T1* is involved. This indicates that P_j is 'bigger' than the cell populations from dataset 1 as part of it is unlabeled. Therefore, we add P_j as a descendant to the root of *T1*. Next, we rewire the involved cell populations from dataset 1 such that they become descendants of P_j (Supplementary Fig. 26C).
3. The root node of *T2* is involved. This indicates that multiple cell populations from dataset 1 are missing in dataset 2. These nodes, however, are already in the tree, so the tree can remain the same (Supplementary Fig. 26D).

Complex

The scenarios described above were all relatively easy. A cell population from one dataset matches either one or multiple cell populations from another. It could also happen, however, that multiple cell populations from dataset 1 match multiple cell populations from dataset 2 (Supplementary Fig. 27). As a consequence, there will a certain place $X_{j,I}$ which is either 1 or 2 and there are two or more non-zero values in the corresponding row *j* and column *i.* Here, we distinguish three different scenarios:

1. The root node of *T1* is involved. We just assume that the boundary should be adjusted and this is automatically done, so we remove this `1' from the table (Supplementary Fig. 27A). If the situation is still complex after the one is removed, we continue to scenario 2 or 3. If not, we treat it as a multiple rows problem as explained above.
2. The root node of *T2* is involved. Again, we just assume that the boundary should be adjusted, so we remove this `1' from the table (Supplementary Fig. 27B). If the situation is still complex after the one is removed, we continue to scenario 3. If not, we treat it as a multiple columns problem as explained above.
3. Multiple leaf/internal nodes of dataset 1 are involved and multiple leaf nodes of dataset 2. We can only solve this if the 'complex' cell population, P_i, of dataset 1 is not a leaf node. Otherwise we are dealing with an impossible scenario which is described below. If the complex node is an internal node, we attach the involved cell populations of dataset 2 as descendants to the complex node (splitting scenario) and attach the involved cell populations of dataset 1, except for P_i, to P_j (Supplementary Fig. 27C).

Impossible

Sometimes, it could be impossible to match the labels from two datasets. Something could have gone wrong during the clustering, e.g. a population 1 and 2 from dataset 1 match population A from dataset 2, but population 2 also matches population C from dataset 2 (Supplementary Fig. 28A). Here, population A and C should be merged into population 2, but population A should also be split into population 1 and 2. Population 2, however, cannot be added to the tree twice.

It could also be that dataset 2 contains labels at a different resolution, e.g. that population B is a subpopulation of population A (Supplementary Fig. 28B). This is not what we assumed and thus impossible to match.

Both scenarios occur when a leaf node from dataset 1 is at a crossing of multiple rows and multiple columns (i.e. a complex situation). An extra difficulty is that there are thus multiple situations that could explain this. All of these situation are not what we desired and thus we call it impossible and do nothing.

Supplementary Note 2

If there is a complex scenario that cannot be solved immediately, matrix *X* will be changed into a strict matrix. In the strict matrix, only reciprocal matches are considered, so all '1's' are turned into '0'. There are some exceptions to this rule.

- A population can never have a reciprocal match with the root, so these '1's' are never removed.
- If a population from a dataset has only one match, it is also never removed. Consider the following example: If population P1 of Dataset 1 is only predicted to be Population Q of Dataset 2, we know that P1 should be a match with Q as it cannot be matched with any other population or with the root. It could be that this match is not reciprocal if population Q has many different subpopulations (e.g. P1, P2, P3, P4). Imagine that population P2 is really big. Almost all cells of population Q will be predicted to be P2 and so the matches with P1 (and P3 and P4) are missed because of the matching threshold. In case there is a complex scenario caused by any other population (maybe P2 or P3 or P4), we still know that P1 is a subpopulation of Q, since that was super clear and didn't cause any complexity.

Supplementary Note 3

Current scRNA-seq data simulators cannot simulate hierarchical data, so we simulated this dataset step by step (Supplementary Fig. 1B).

First, we simulated the expression of 3,000 genes for 9,000 cells. For this simulation, the cells were divided into three groups. The 3,000 simulated genes represent genes that are differentially expressed between the cell populations at a low resolution, so for example B cells vs. T cells. Next, we simulated *another* 3,000 genes for the *same* 9,000 cells. Now, the cells were divided into five groups. Here, the differentially expressed genes represent genes that distinguish cell populations at a slightly higher resolution, so for example CD4+ T cells vs. CD8+ T cells. We repeated this step for another set of 3,000 genes, but now there were six populations. The third dataset represents the highest resolution, so for instance CD4+ memory T cells vs. CD4+ naïve T cells.

Together this resulted in a dataset of 9,000 cells and 9,000 genes. The cells were labeled at three resolutions. There was some inconsistency between the labels at the different resolutions (e.g. some cells were labeled as 'Group12', 'Group3', 'Group3'). We removed these cells from the dataset, which resulted in a final dataset of 8,839 cells and 9,000 genes.

Supplementary Figures

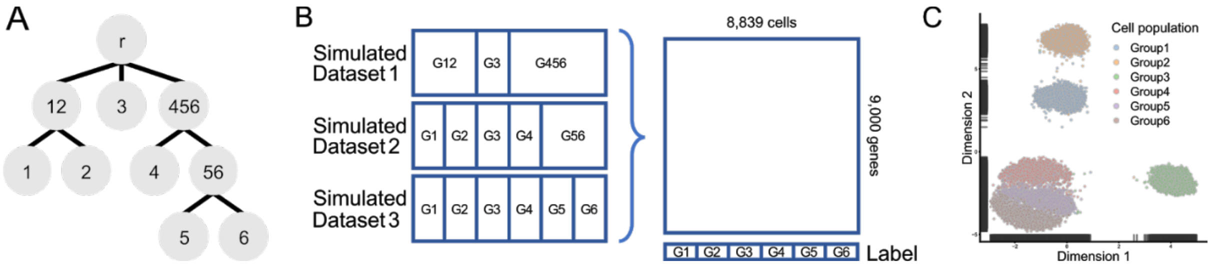

Supplementary Fig. 1 (A) Classification tree for the simulated dataset. (B) We simulated three datasets separately and concatenated them in one dataset. The labels and their proportion are indicated in the simulated datasets. (C) UMAP of the final dataset.

A

B

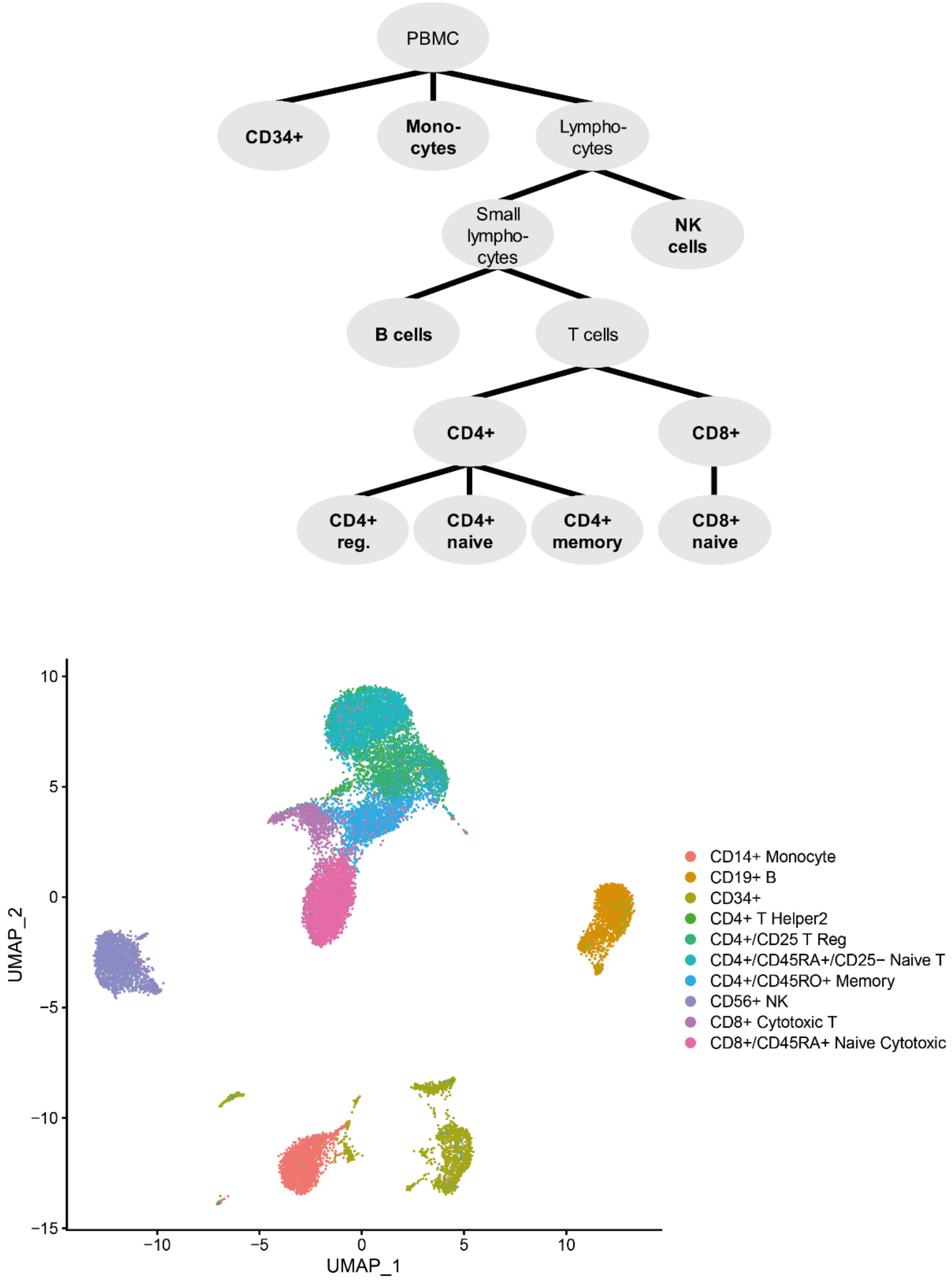

Supplementary Fig. 2 (A) Classification tree for the PBMC-FACS dataset. Bold names indicate cell populations that exist in our dataset. (B) UMAP embedding of the PBMC-FACS data

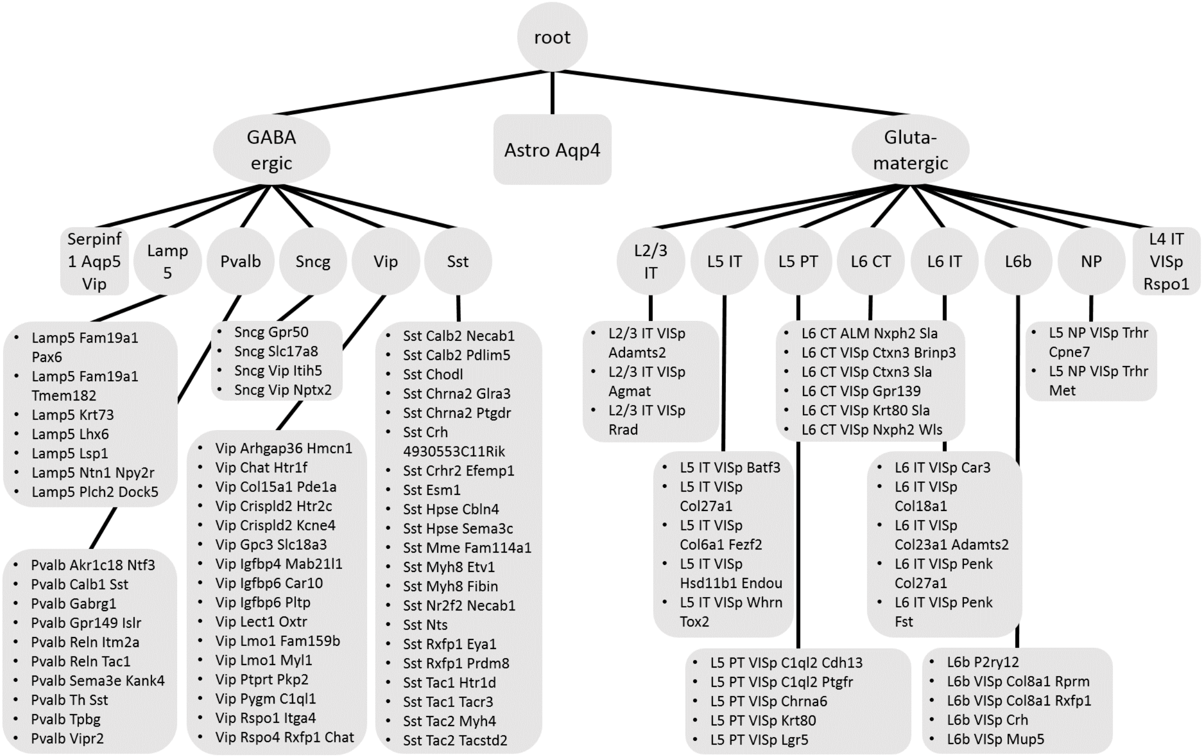

Supplementary Fig. 3 Classification tree of the AMB dataset. The circular and rectangle nodes indicate internal and leaf nodes respectively. If multiple leaf nodes are descendants of the same internal node, they are placed in the same rectangle.

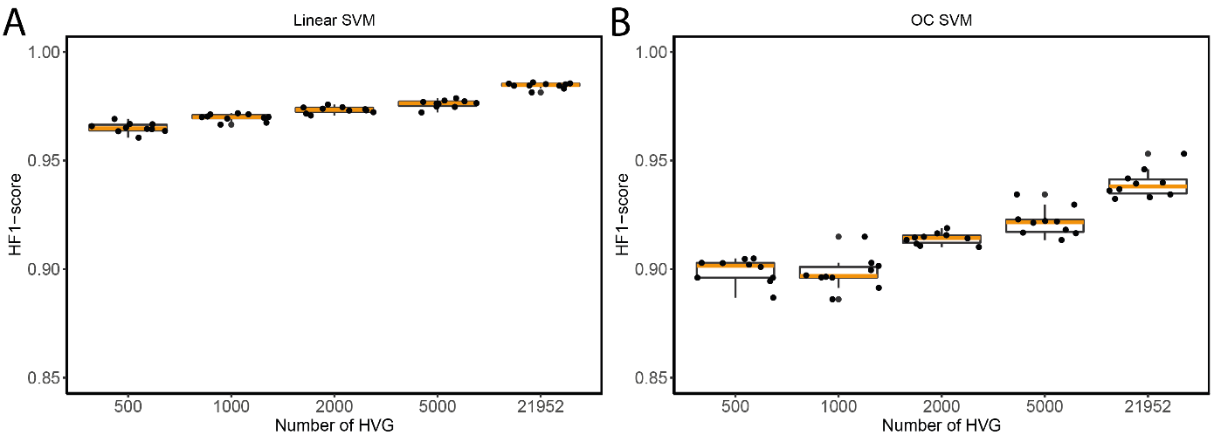

Supplementary Fig. 4 Effect of selection HVG on the classification performance on the PBMC-FACS dataset when using the (A) linear SVM and (B) one-class SVM during cross-validation (*n* = 10). In the boxplots, the middle (orange) line represents the median, the lower and upper hinge represent the first and third quartiles, and the lower and upper whisker represent the values no further than 1.5 inter-quartile range away from the lower and upper hinge respectively.

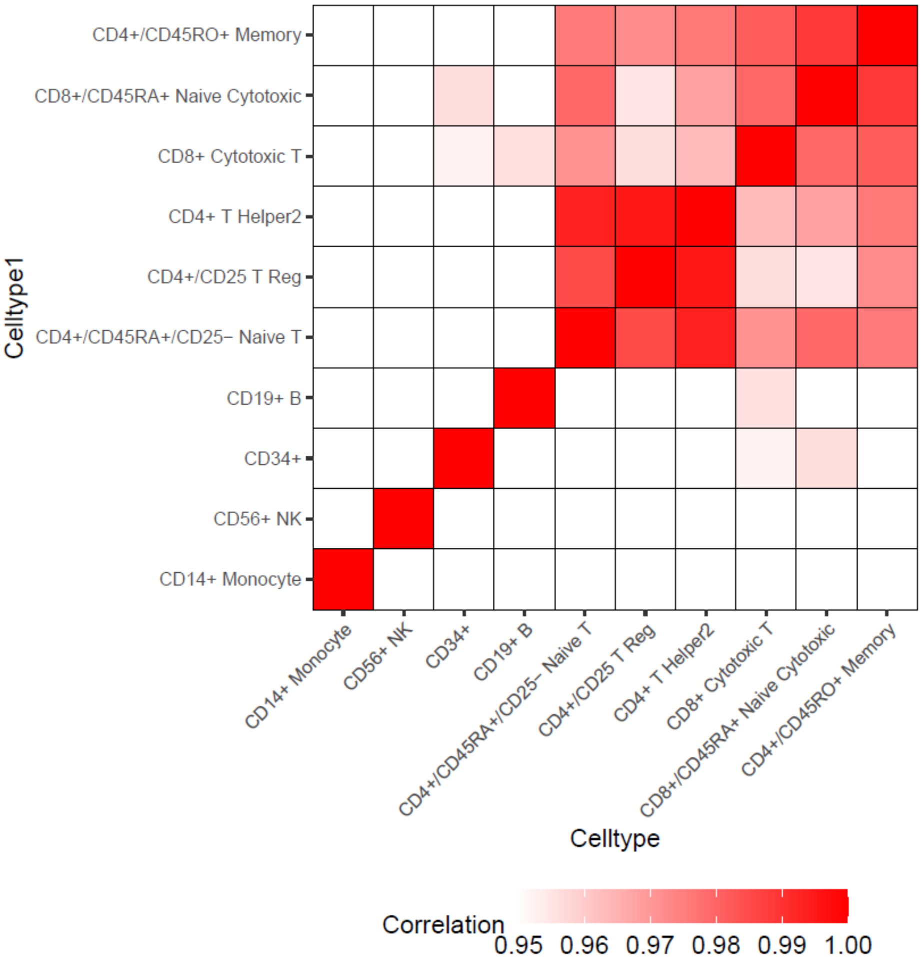

Supplementary Fig. 5 Pearson correlation between the cell population in the PBMC-FACS dataset.

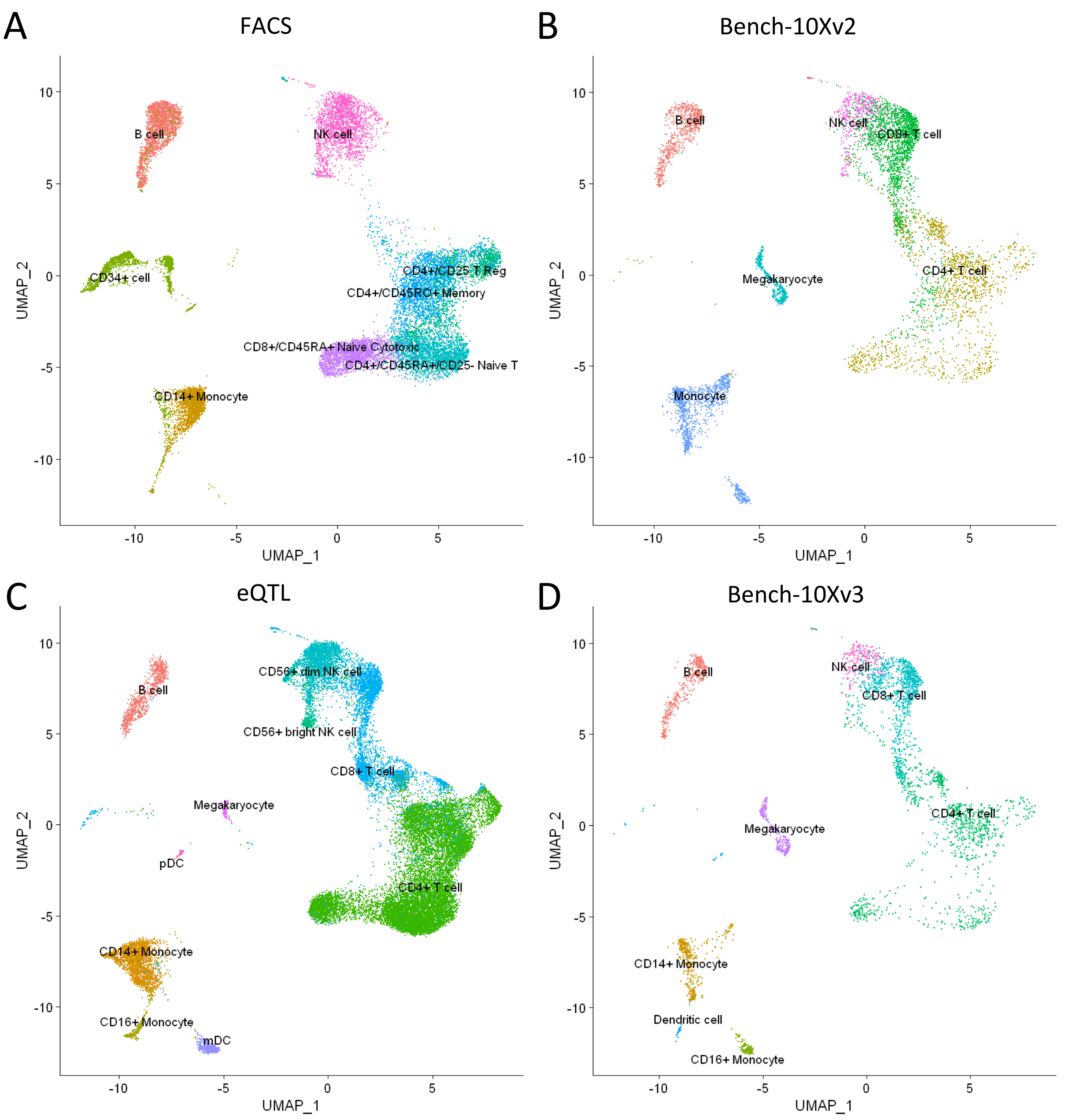

Supplementary Fig. 6 UMAPs showing the cell populations of the (A) FACS, (B) Bench-10Xv2, (C) eQTL, (D) Bench-10Xv3 datasets after integration with Seurat.

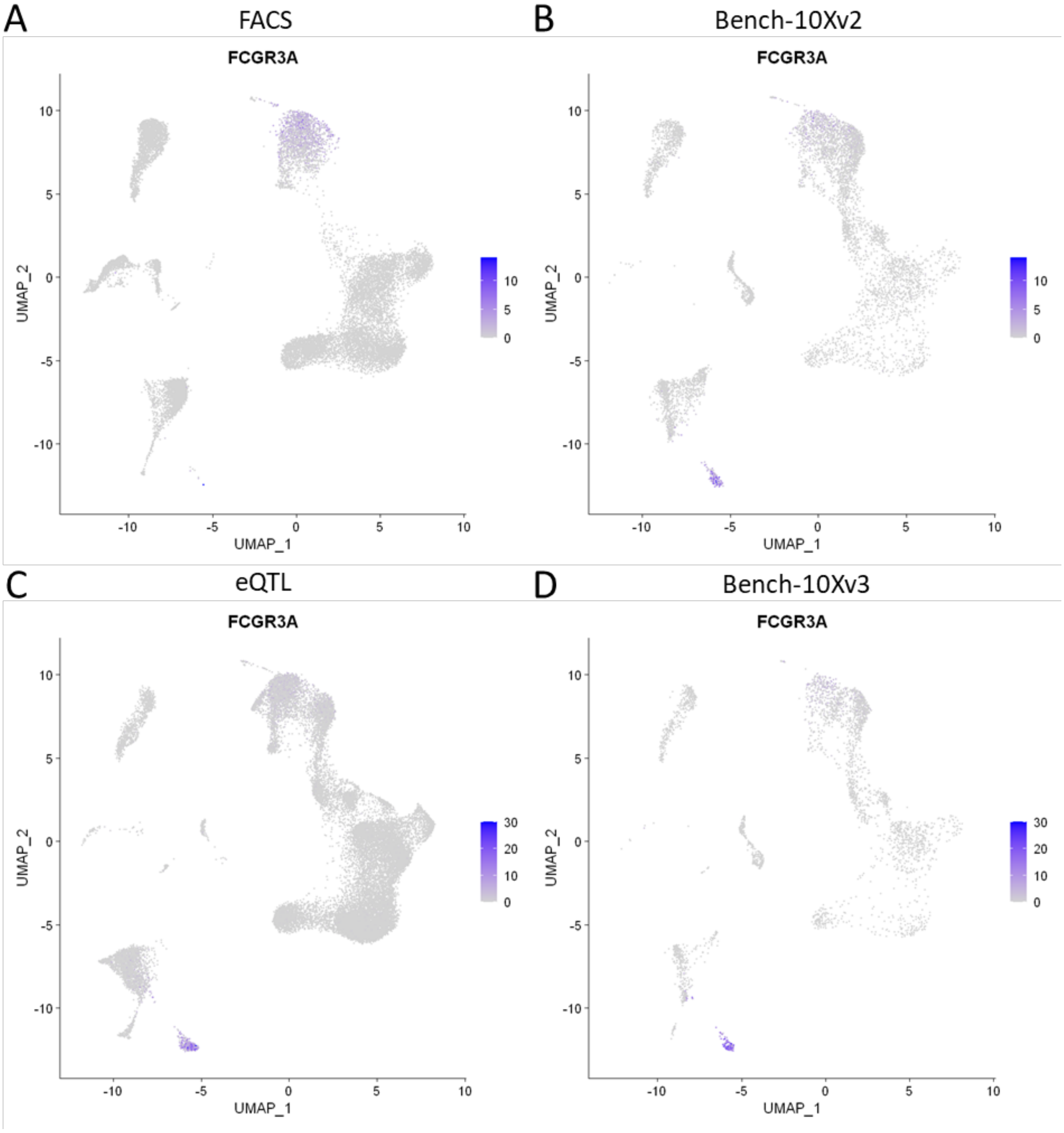

Supplementary Fig. 7 UMAPs showing the expression of *FCGR3A* in the PBMC datasets. We visualized the original counts of *FCGR3A,* so before integration. *FCGR3A* is a marker gene for CD16+ monocytes. In the eQTL dataset, *FCGR3A* is expressed in the cells labeled as mDC (Supplementary Fig. 6).

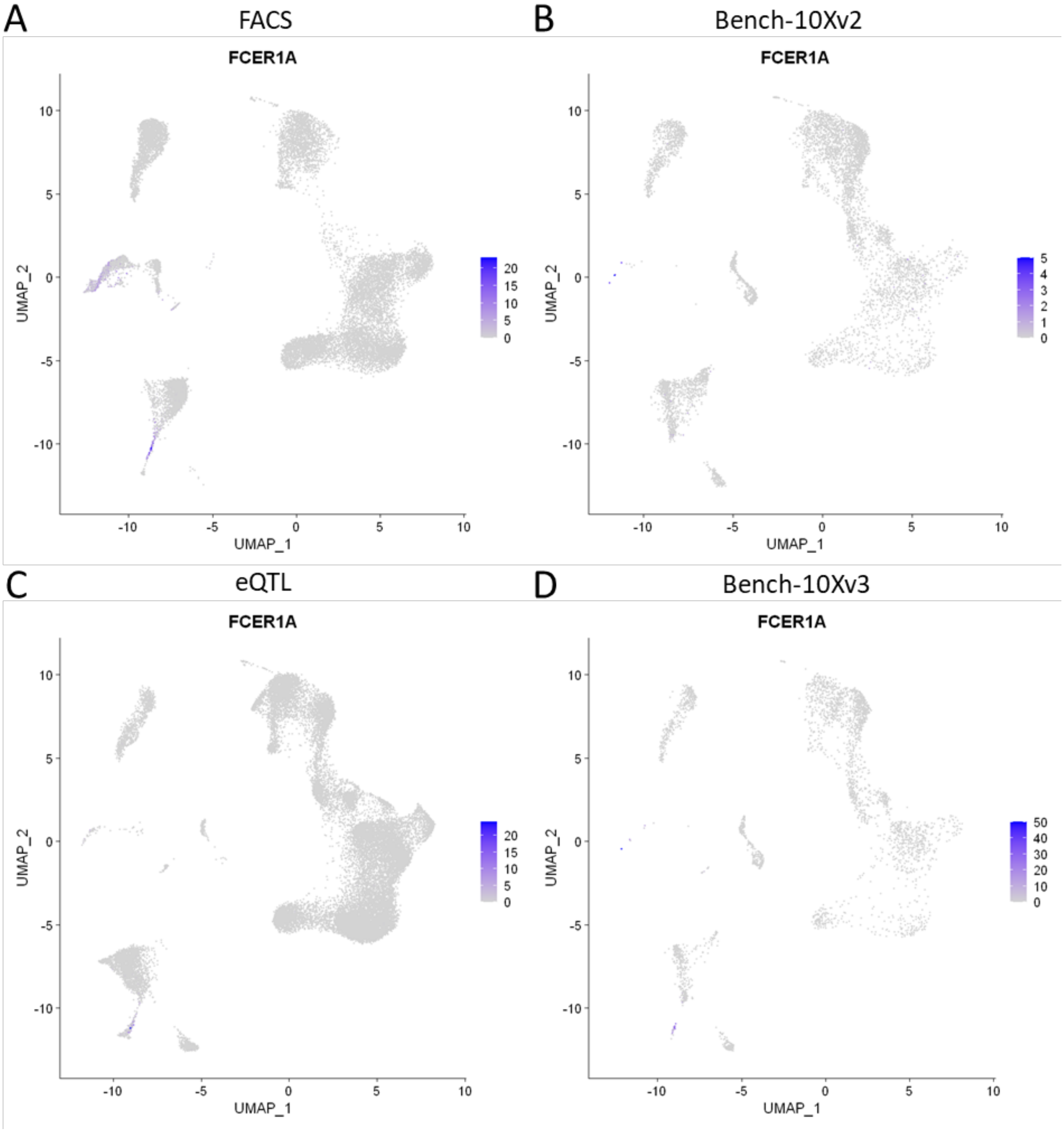

Supplementary Fig. 8 UMAPs showing the expression of *FCER1A* in the PBMC datasets. We visualized the original counts of *FCER1A,* so before integration. *FCER1A* is a marker gene for mDC. In the eQTL dataset, *FCER1A* is lowly expressed in the cells labeled as CD16+ monocytes (Supplementary Fig. 6). In the FACS dataset, part of the cells labeled as CD14+ monocytes also expressed *FCER1A.*

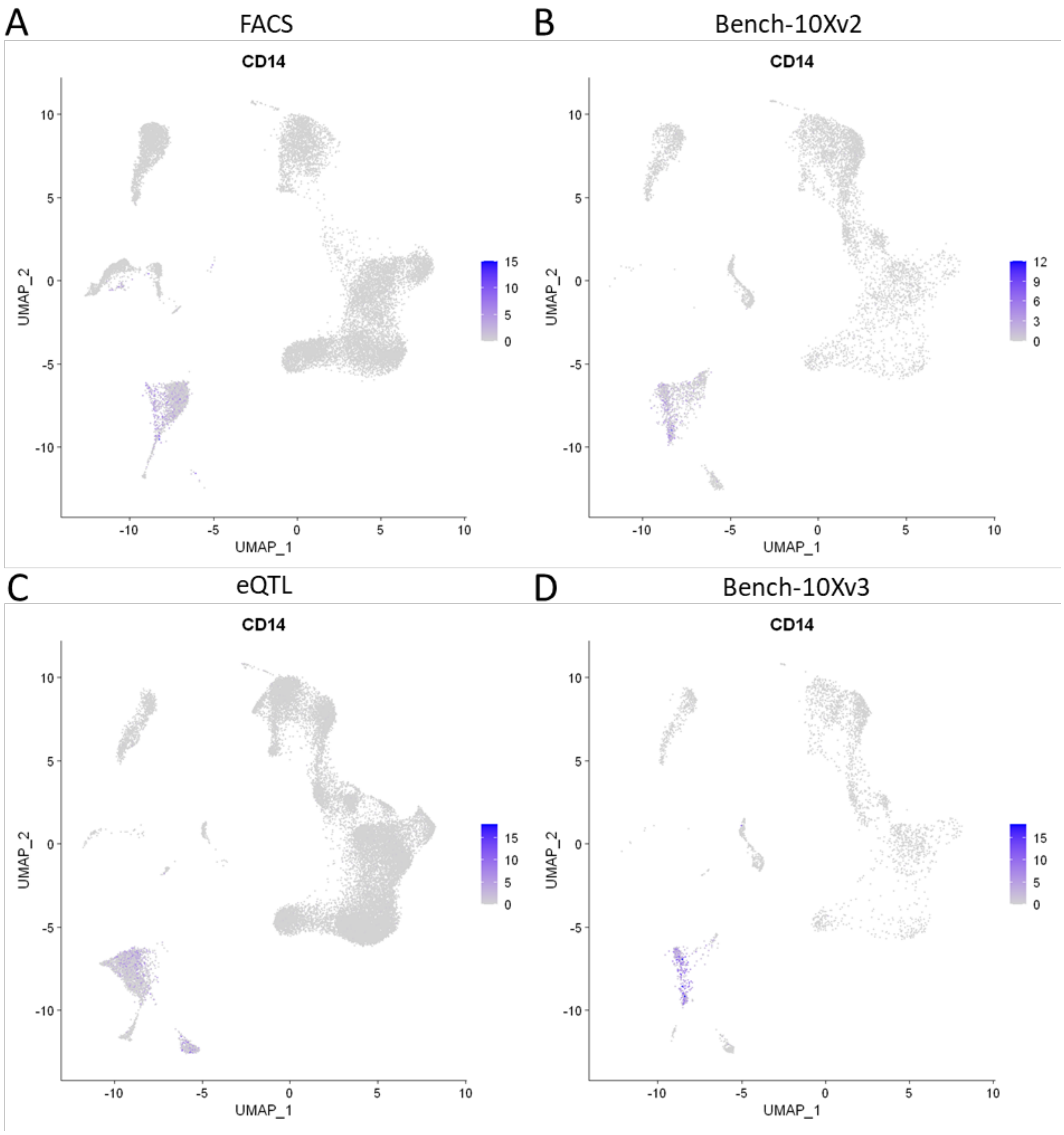

Supplementary Fig. 9 UMAPs showing the expression of *CD14* in the PBMC datasets. We visualized the original counts of *CD14,* so before integration. *CD14* is a marker gene for CD14+ monocytes. In the FACS dataset, part of the cells labeled as CD14+ monocytes express no CD14+ (Supplementary Fig. 6).

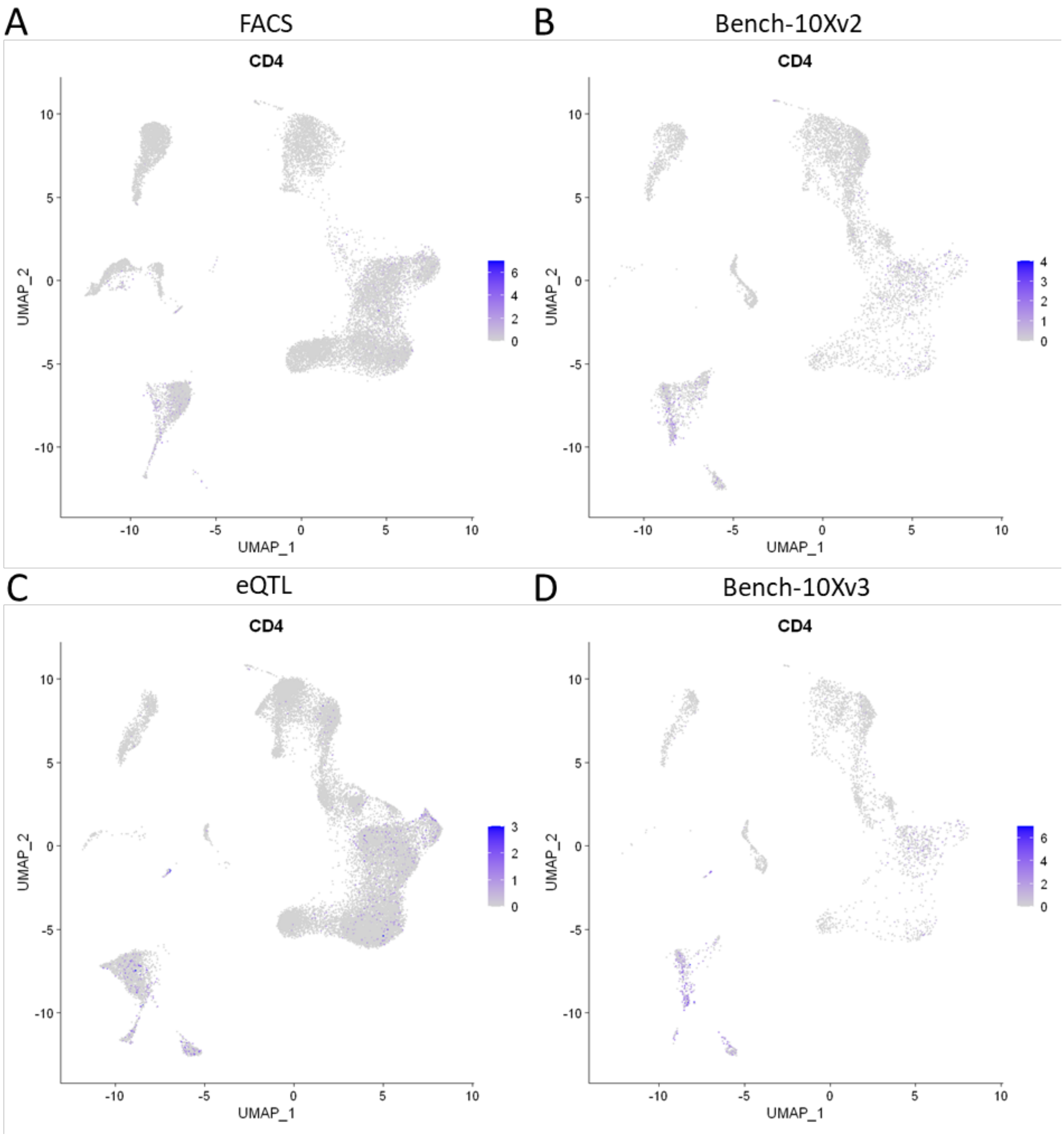

Supplementary Fig. 10 UMAPs showing the expression of *CD4* in the PBMC datasets. We visualized the original counts of *CD4,* so before integration. *CD4* is a marker gene for CD4+ T-cells and monocytes. In the eQTL and Bench-10Xv2 dataset, *CD4* is only lowly expressed in the CD4+ T-cells at the right of the cluster and expression is missing at the bottom-left part of the cluster (Supplementary Fig. 6).

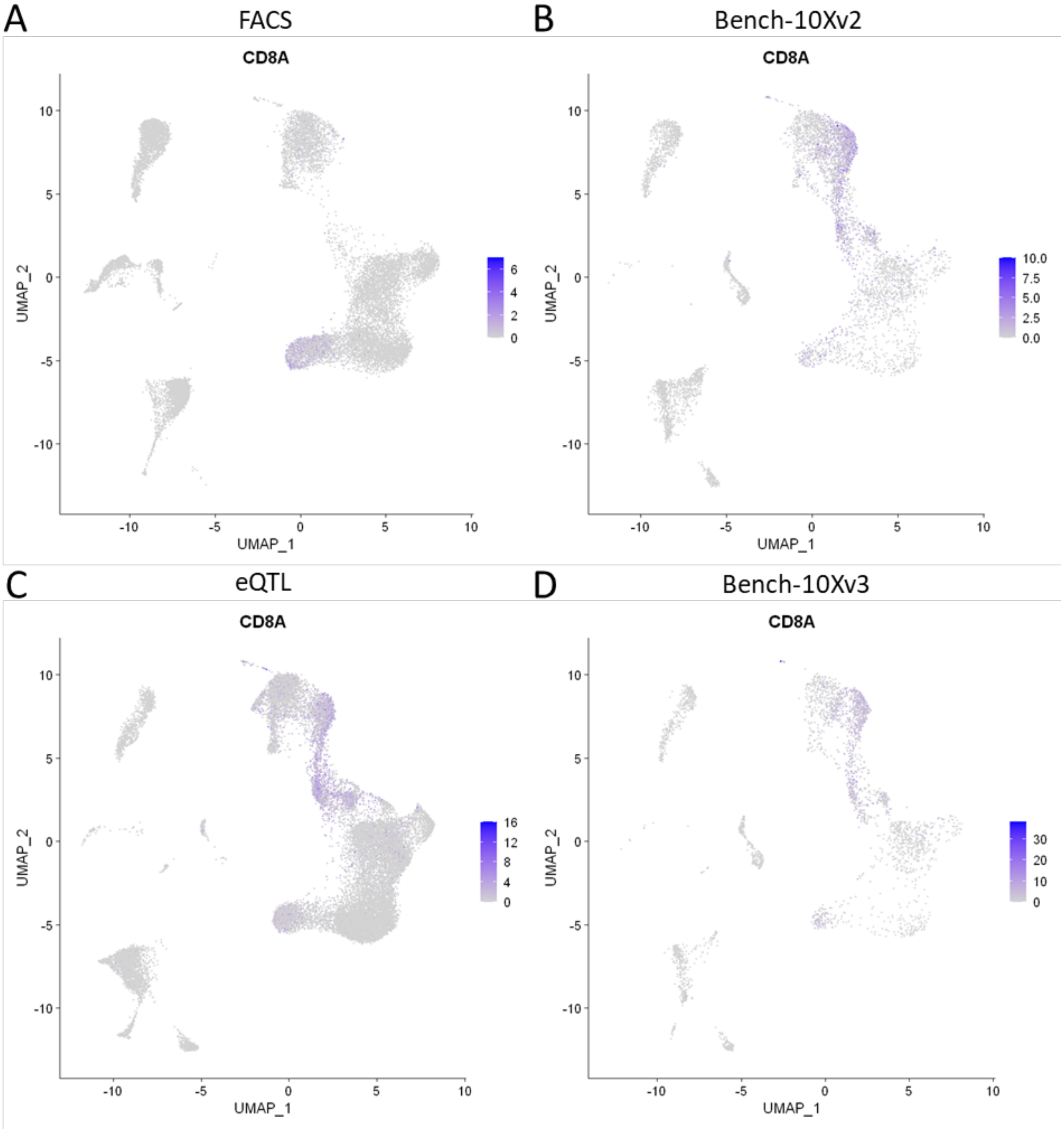

Supplementary Fig. 11 UMAPs showing the expression of *CD8A* in the PBMC datasets. We visualized the original counts of *CD8A,* so before integration. *CD8A* is a marker gene for CD8+ T-cells. In the eQTL and Bench-10Xv2 dataset, *CD8A* is also expressed in the CD4+ T-cells at the bottom-left part of the cluster (Supplementary Fig. 6).

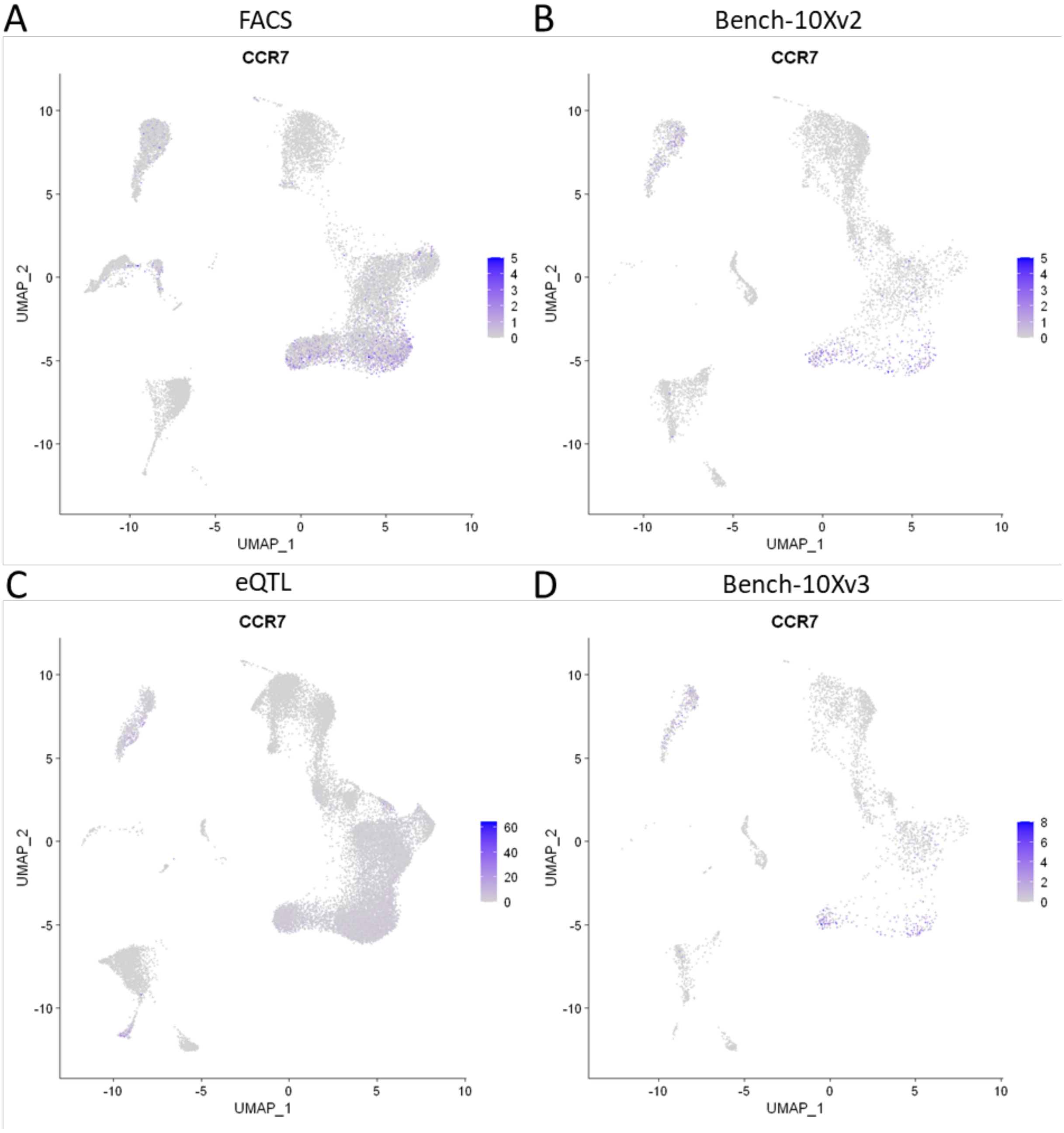

Supplementary Fig. 12 UMAPs showing the expression of *CCR7* in the PBMC datasets. We visualized the original counts of *CCR7,* so before integration. *CCR7* is a marker gene for naive T-cells.

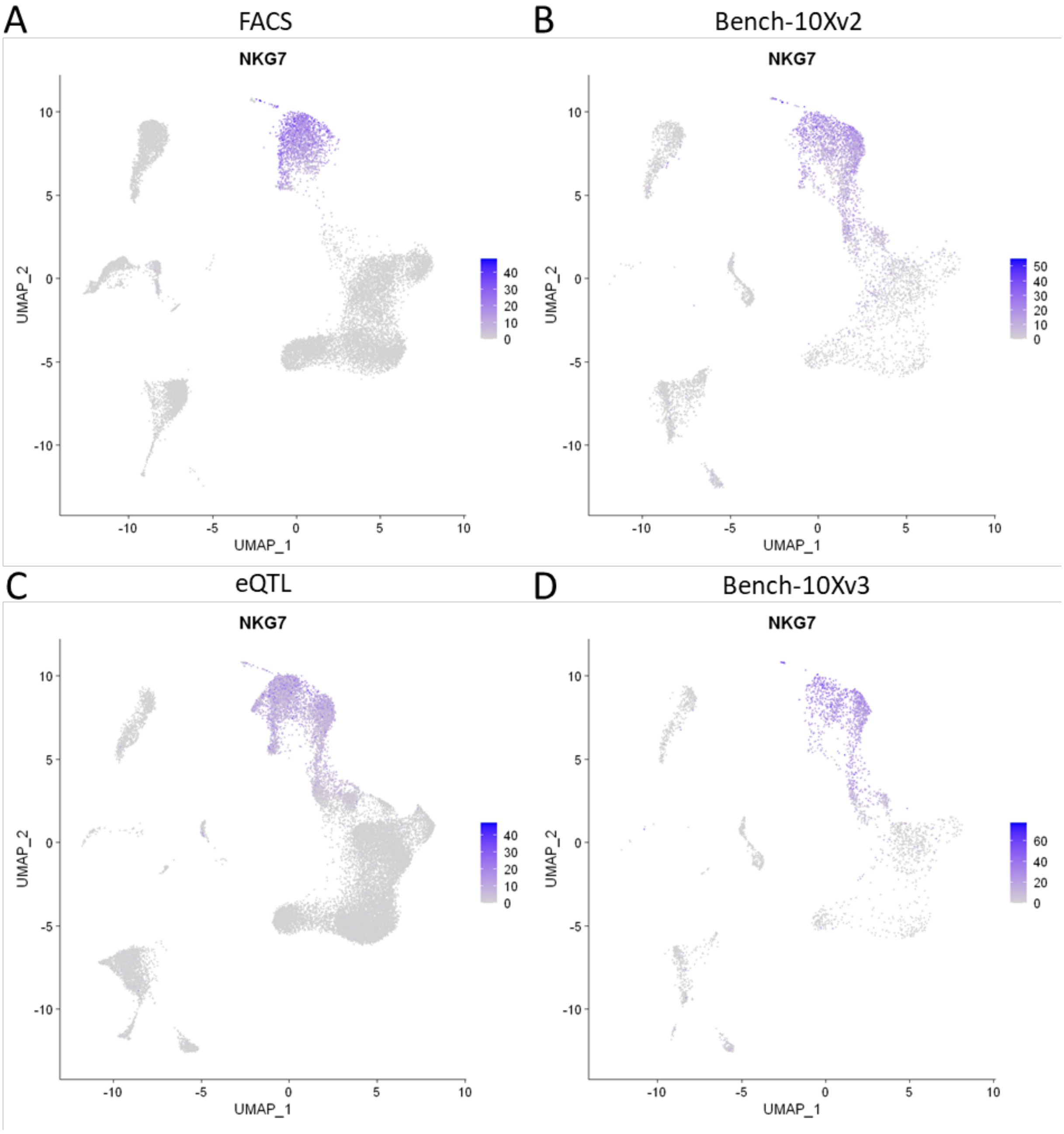

Supplementary Fig. 13 UMAPs showing the expression of *NKG7* in the PBMC datasets. We visualized the original counts of *NKG7,* so before integration. *NKG7* is a marker gene for NK cells and cytotoxic T-cells.

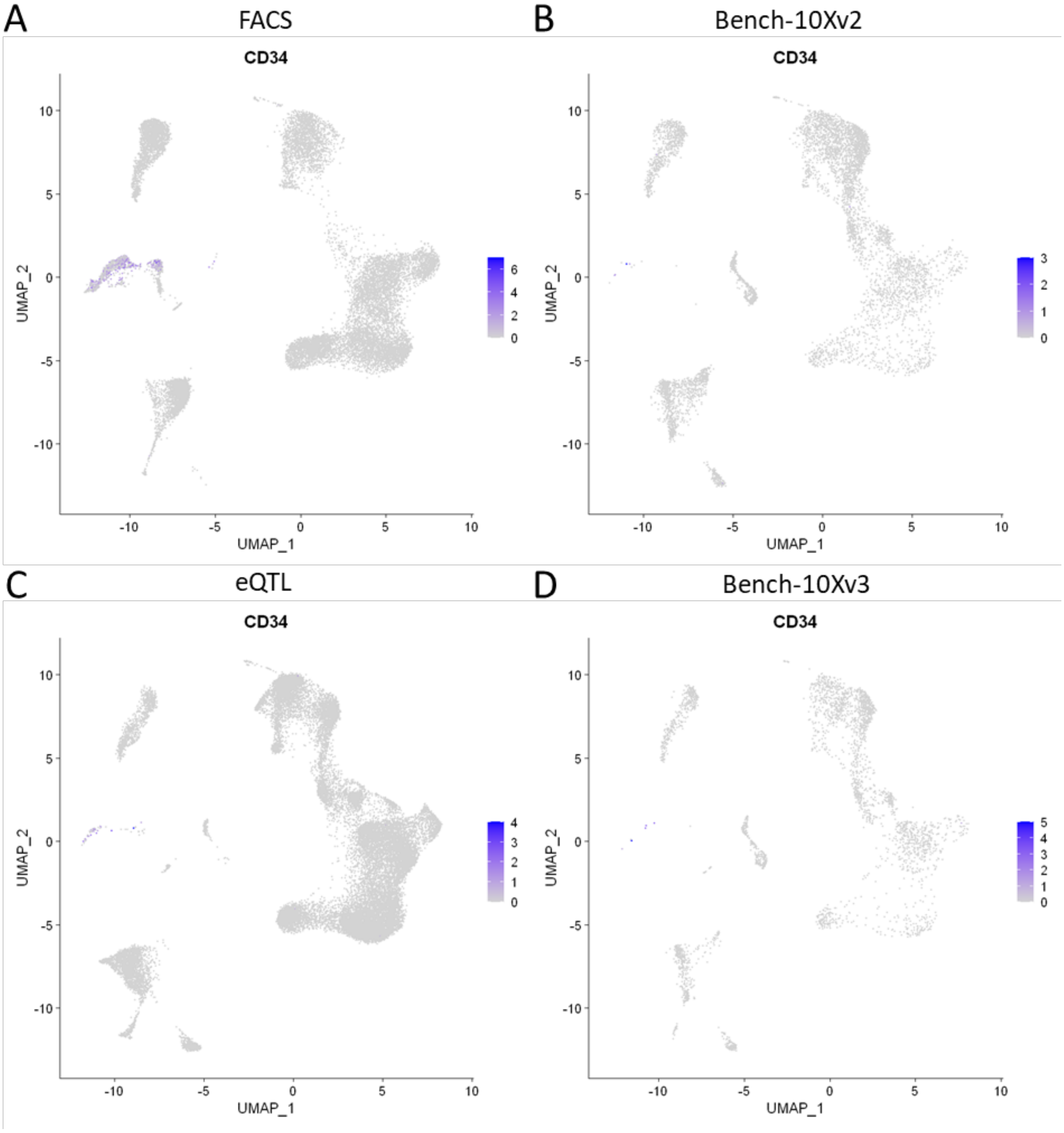

Supplementary Fig. 14 UMAPs showing the expression of *CD34* in the PBMC datasets. We visualized the original counts of *CD34,* so before integration. *CD34* is a marker gene for CD34+ cells.

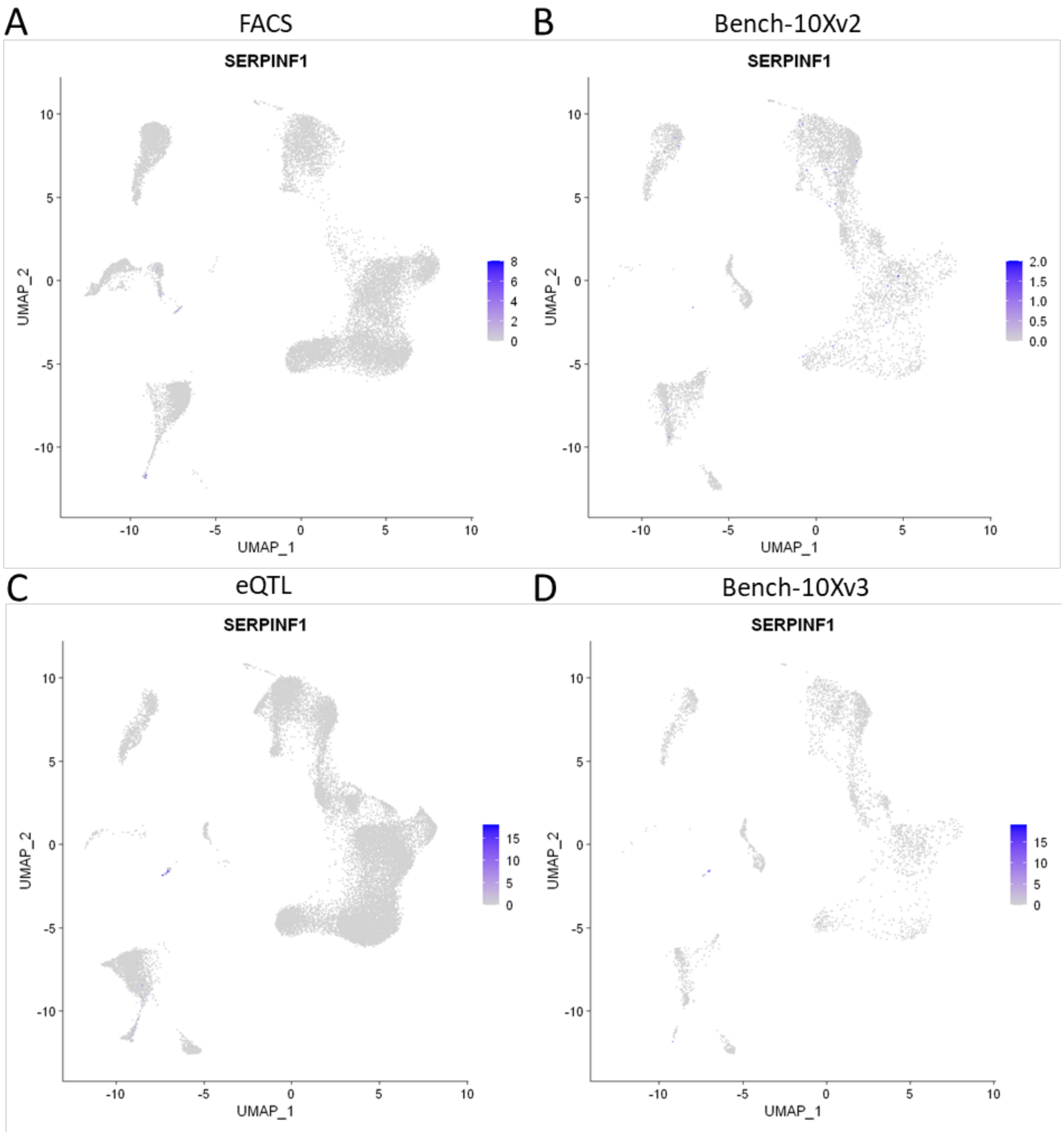

Supplementary Fig. 15 UMAPs showing the expression of *SERPINF1* in the PBMC datasets. We visualized the original counts of *SERPINF1,* so before integration. *SERPINF1* is a marker gene for pDC.

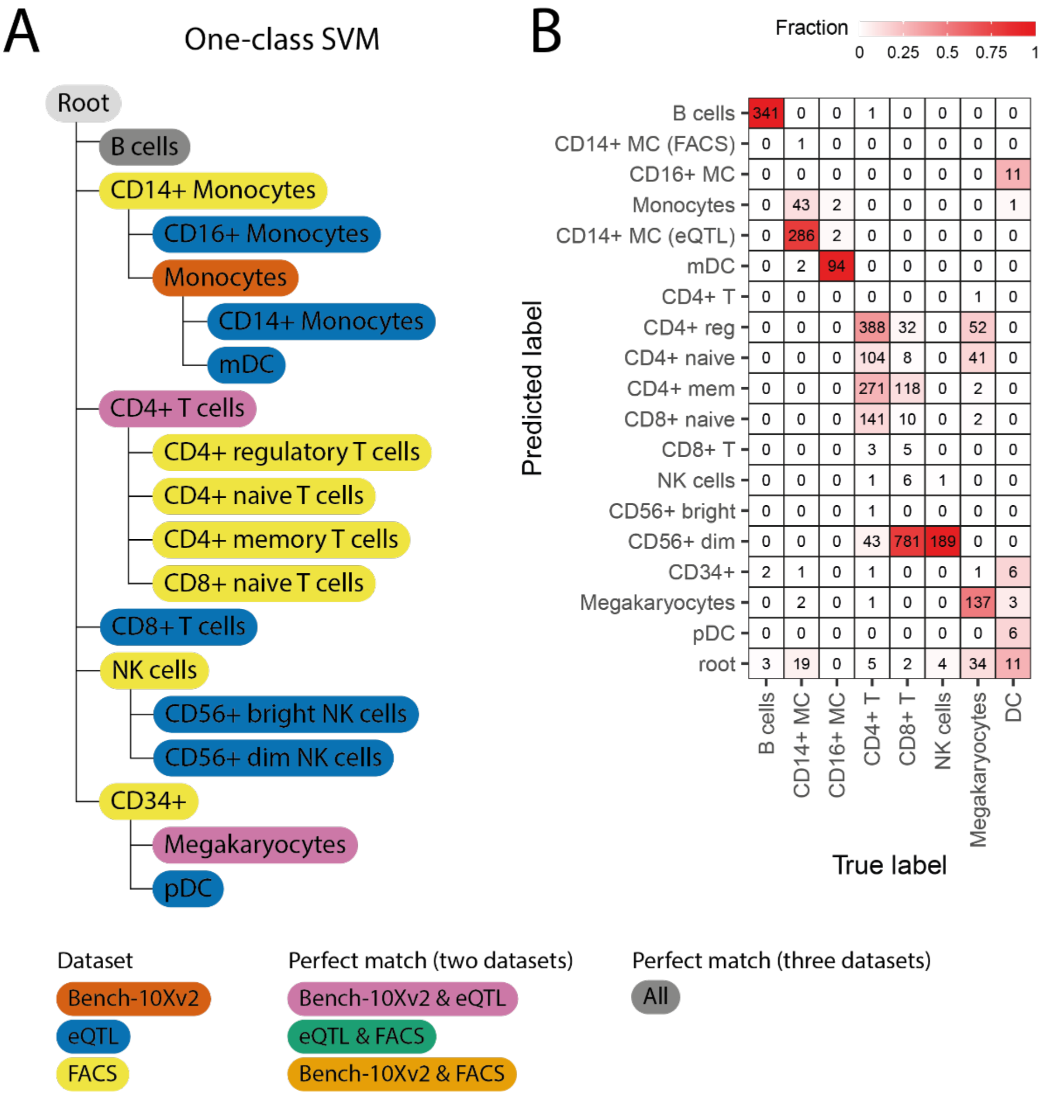

Supplementary Fig. 16 (A) Constructed classification tree when using a one-class SVM during the PBMC inter-dataset experiment. The color of a node represents the dataset(s) of the cell population. If a color refers to multiple datasets, this indicates that the populations from these datasets had a perfect match. The NK and CD8+ T-cell populations from the PBMC-Bench10Xv2 are missing from the tree. (B) Confusion matrix when using the constructed classification tree to predict the labels of PBMC-Bench10Xv3.

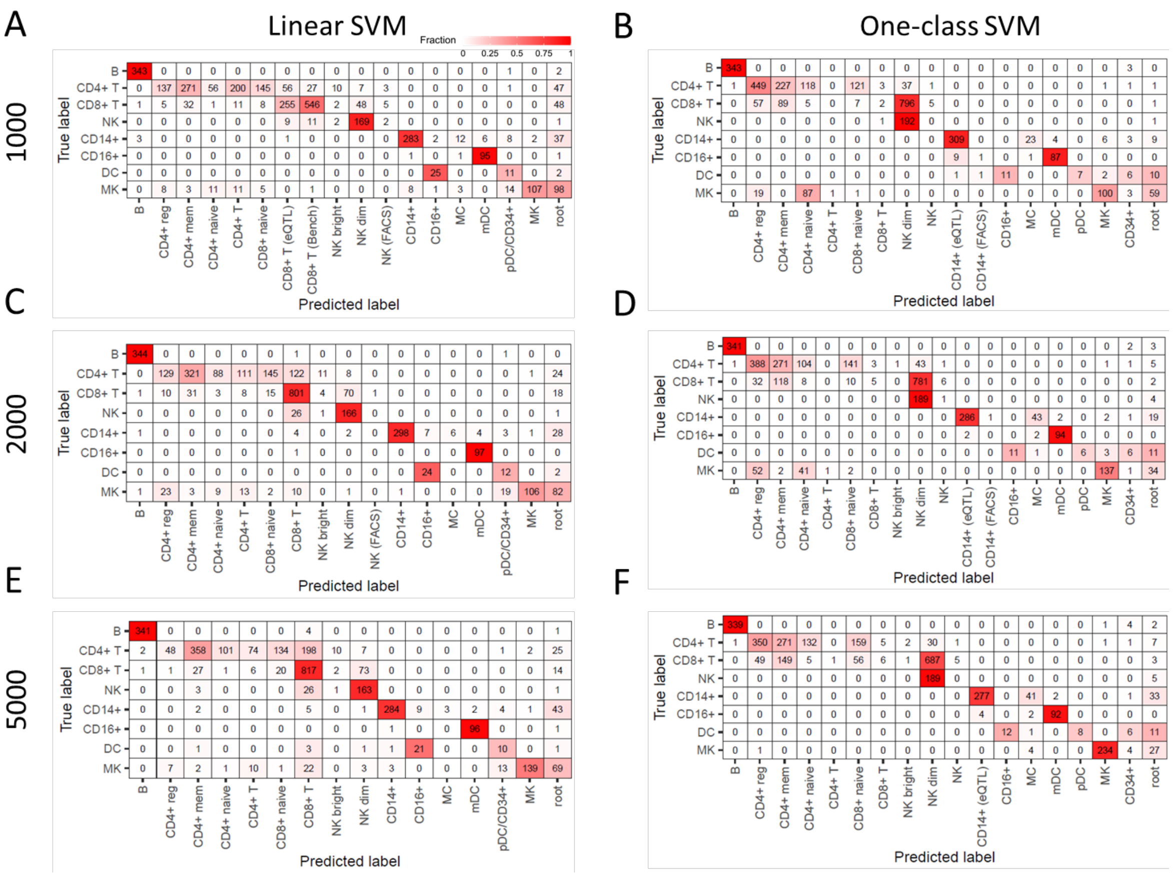

Supplementary Fig. 17 Confusion matrix when using the constructed classification tree to predict the labels of PBMC-Bench10Xv3 with linear and one-class SVM (columns) when varying the number of anchors to integrate the dataset (rows).

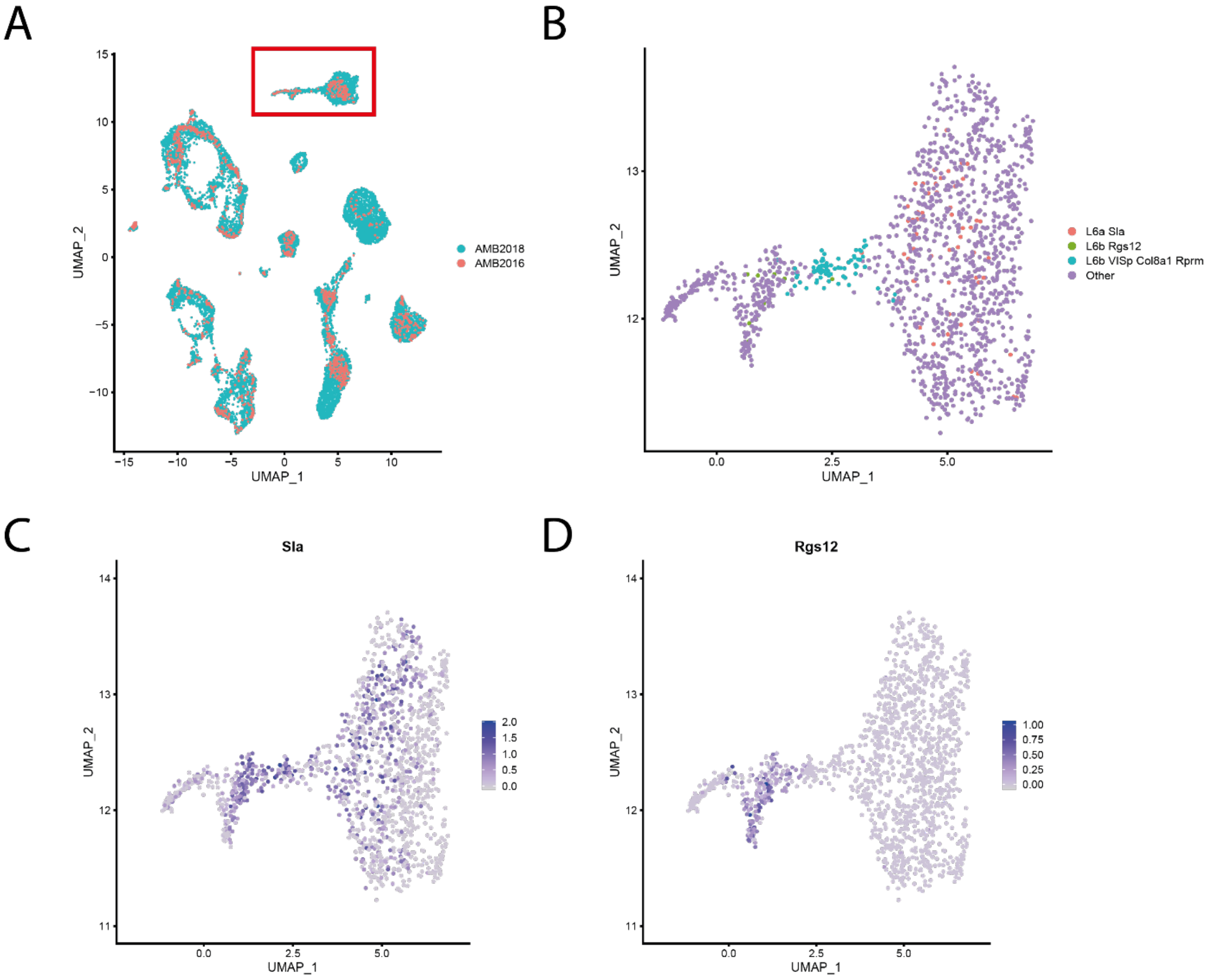

Supplementary Fig. 18 (A) UMAP embedding showing the AMB2016 and AMB2018 datasets after data integration. The red rectangle shows the cells we zoomed in on to visualize the expression of marker genes. (B) Different cell populations within this part of the UMAP. (C-D) Expression of *Sla* and *Rgs12.* The 'L6b VISp Col8a1 Rprm' population does not express *Rgs12,* but does express *Sla,* which support the match with 'L6a Sla' instead of 'L6b Rgs12'.

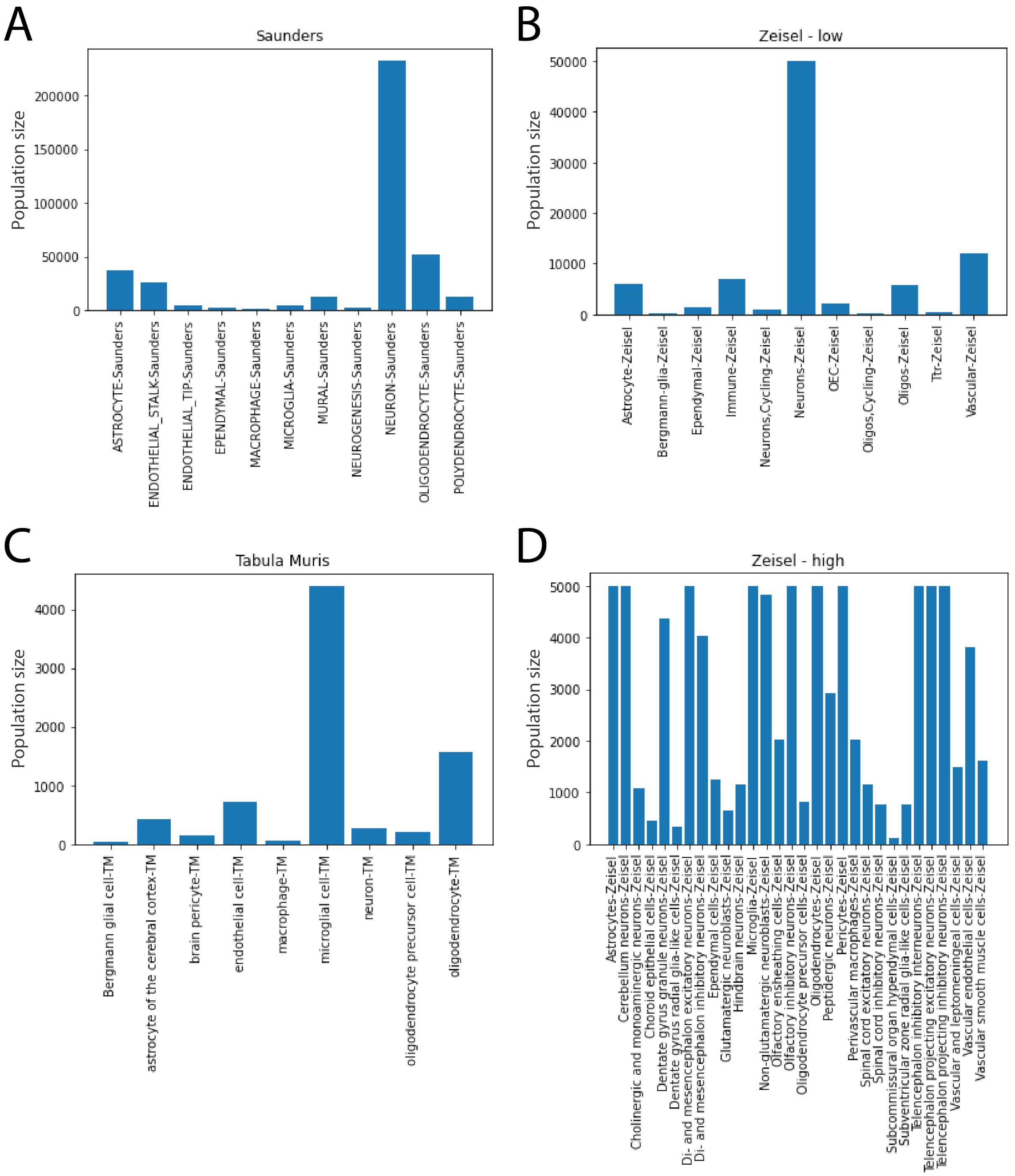

Supplementary Fig. 19 Size of the cell populations in the (A) Saunders, (B) Zeisel – low resolution, (C) Tabula Muris, and (D) Zeisel – high resolution dataset. When comparing the size of the difference populations, notice the different scales on the y-axis.

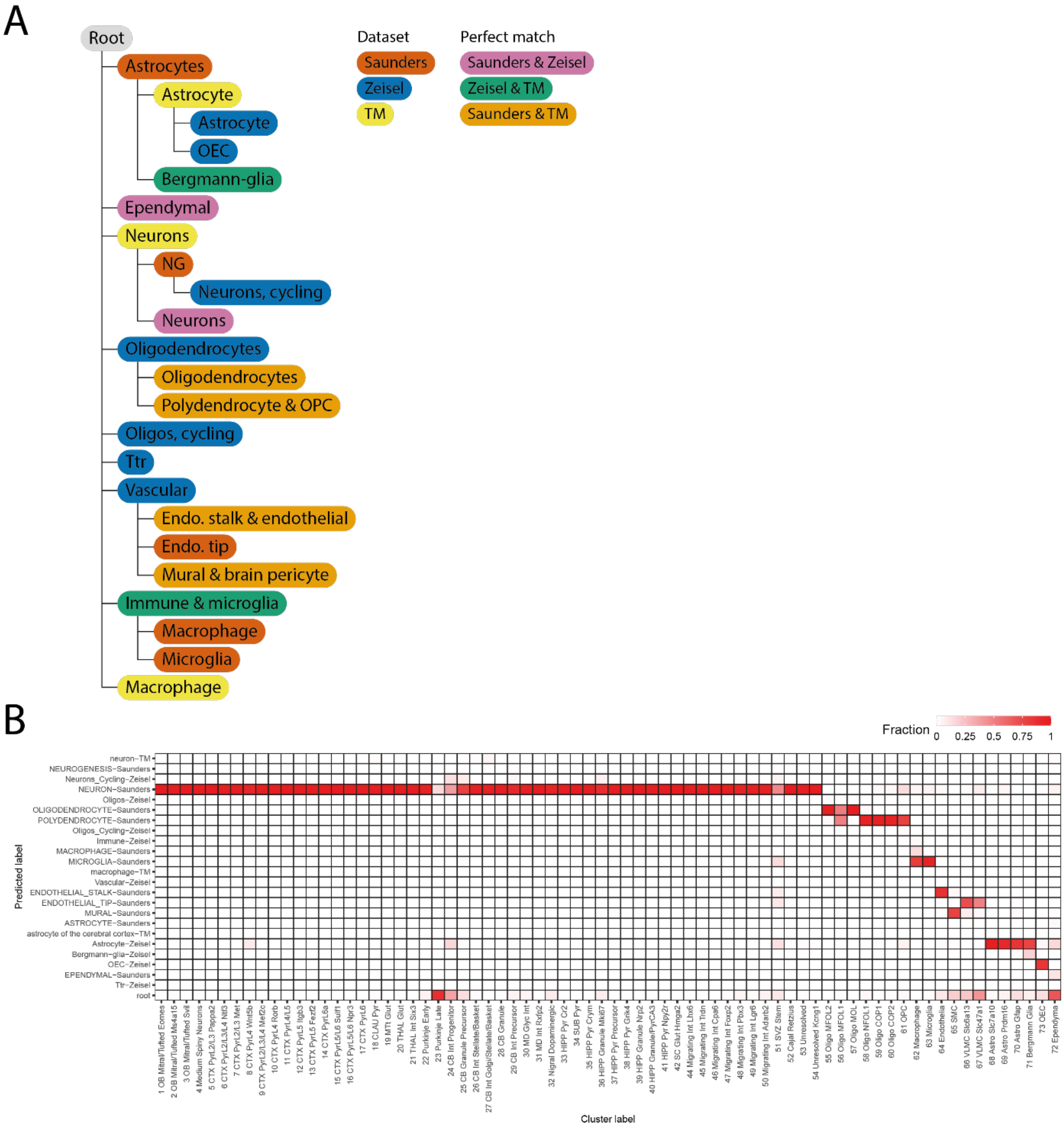

Supplementary Fig. 20 (A) Learned hierarchy when applying *scHPL* with a one-class SVM to the Saunders, Zeisel (low resolution), and Tabula Muris dataset. (B) Confusion matrix when using the learned classification tree to predict the labels of the Rosenberg dataset.

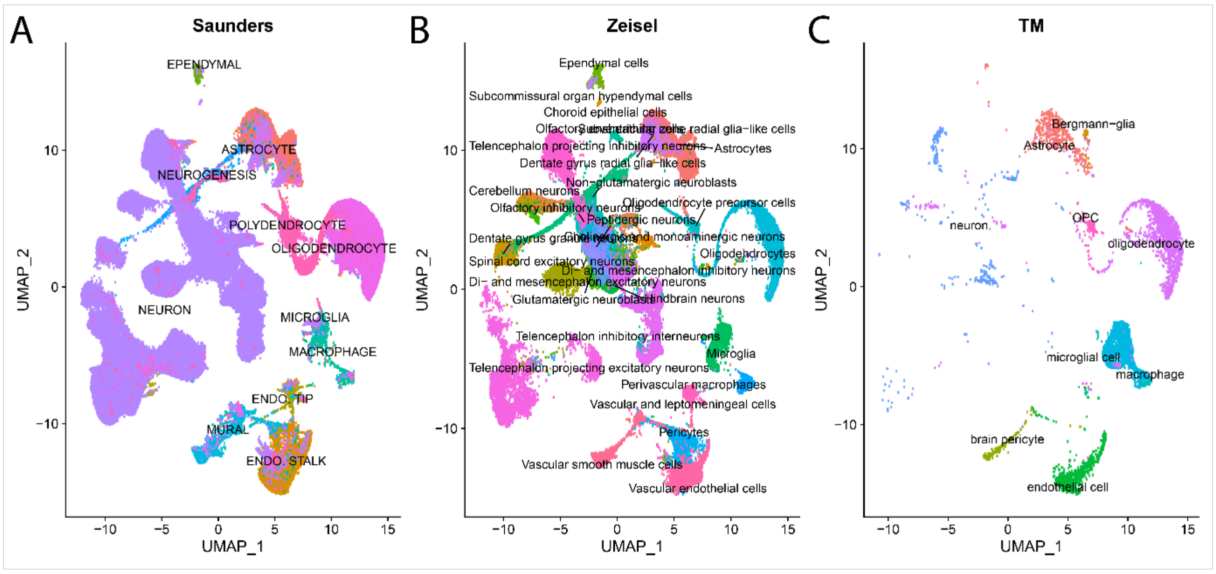

Supplementary Fig. 21 UMAP embeddings of (A) Saunders, (B) Zeisel (high resolution), (C) Tabula Muris after data integration.

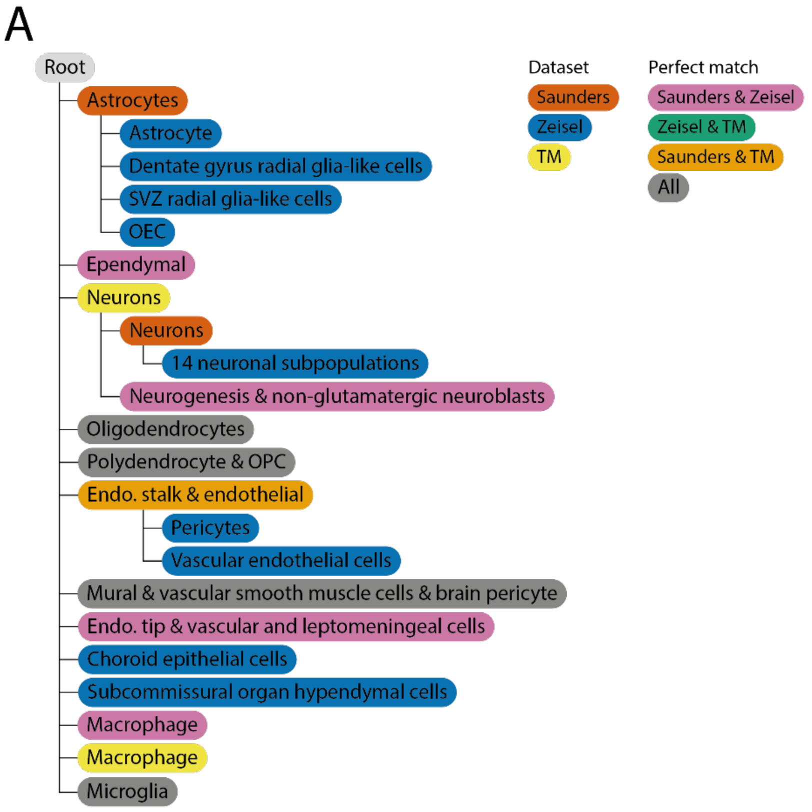

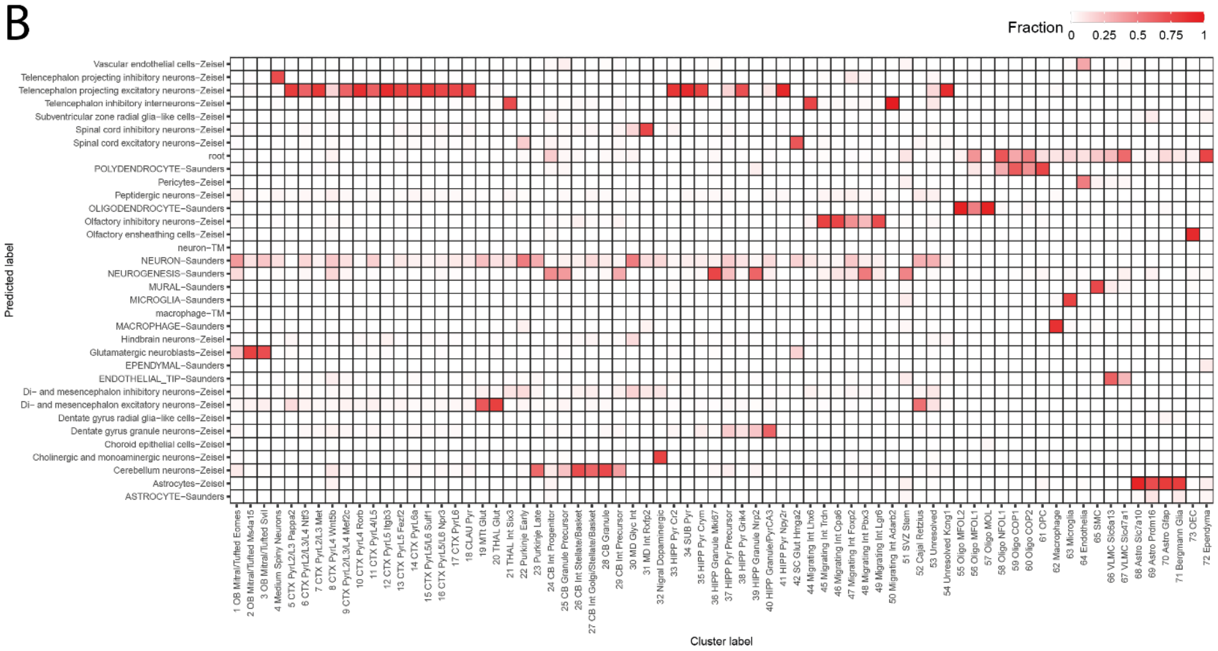

Supplementary Fig. 22 (A) Learned hierarchy when applying *scHPL* with a linear SVM to the Saunders, Zeisel (high resolution), and Tabula Muris dataset. The 14 neuronal populations in the tree include: olfactory inhibitory neurons, cholinergic and monoaminergic neurons, glutamatergic neuroblasts, di- and mesencephalon inhibitory neurons, cerebellum neurons, peptidergic neurons, spinal cord inhibitory neurons, di- and mesencephalon excitatory neurons, spinal cord excitatory neurons, telencephalon projecting excitatory neurons, hindbrain neurons, dentate gyrus granule neurons, telencephalon inhibitory interneurons, and telencephalon projecting inhibitory neurons. Bergmann glia (TM) and astrocytes (TM) are missing from the tree. (B) Confusion matrix when using the learned classification tree to predict the labels of the Rosenberg dataset.

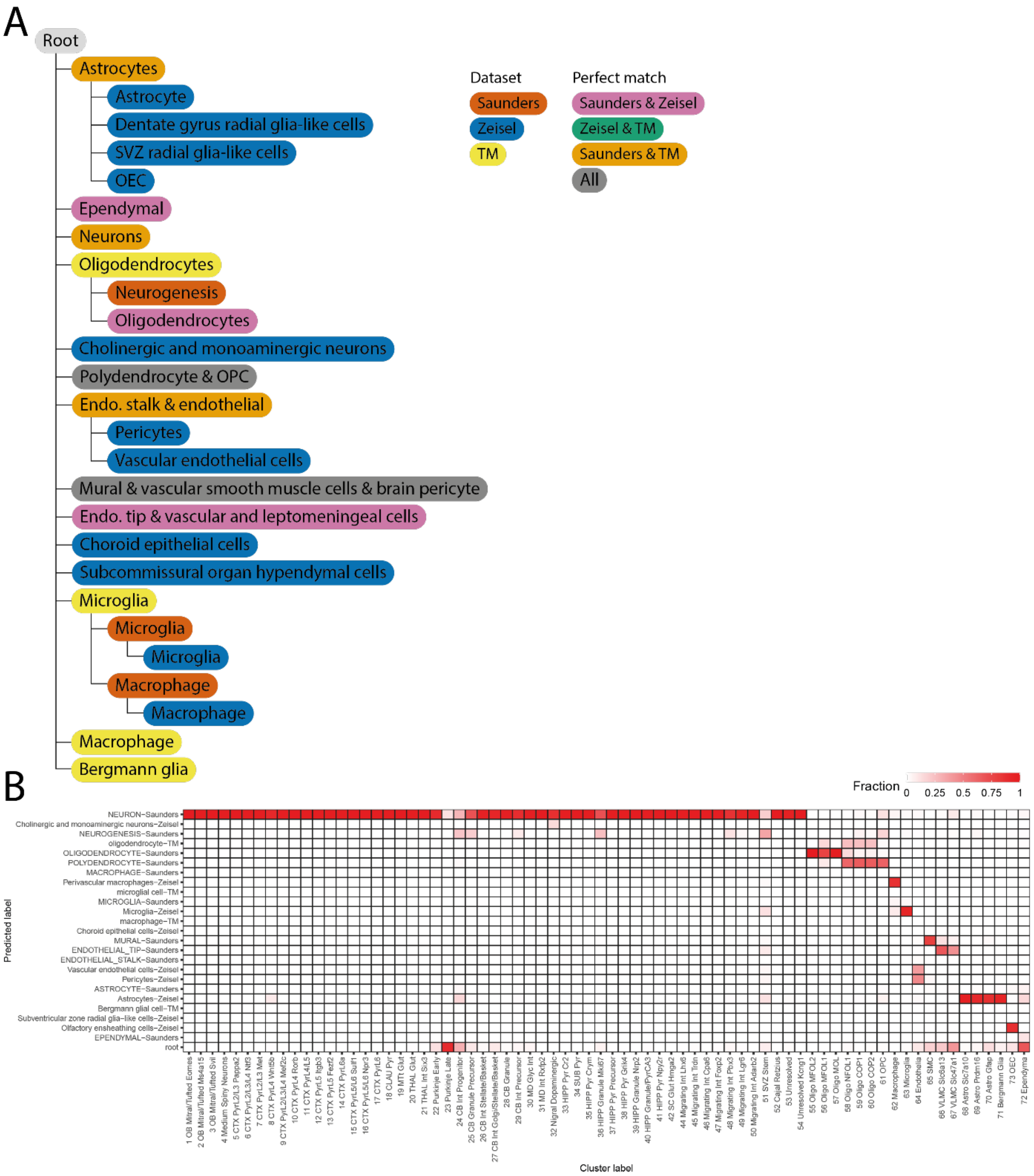

Supplementary Fig. 23 Result one-class SVM on the high resolution. Almost all neuronal cell populations are missing. Fourteen neuronal populations are missing from the tree: olfactory inhibitory neurons, glutamatergic neuroblasts, non-glutamatergic neuroblasts, di- and mesencephalon inhibitory neurons, cerebellum neurons, peptidergic neurons, spinal cord inhibitory neurons, di- and mesencephalon excitatory neurons, spinal cord excitatory neurons, telencephalon projecting excitatory neurons, hindbrain neurons, dentate gyrus granule neurons, telencephalon inhibitory interneurons, and telencephalon projecting inhibitory neurons. (B) Confusion matrix when using the learned classification tree to predict the labels of the Rosenberg dataset.

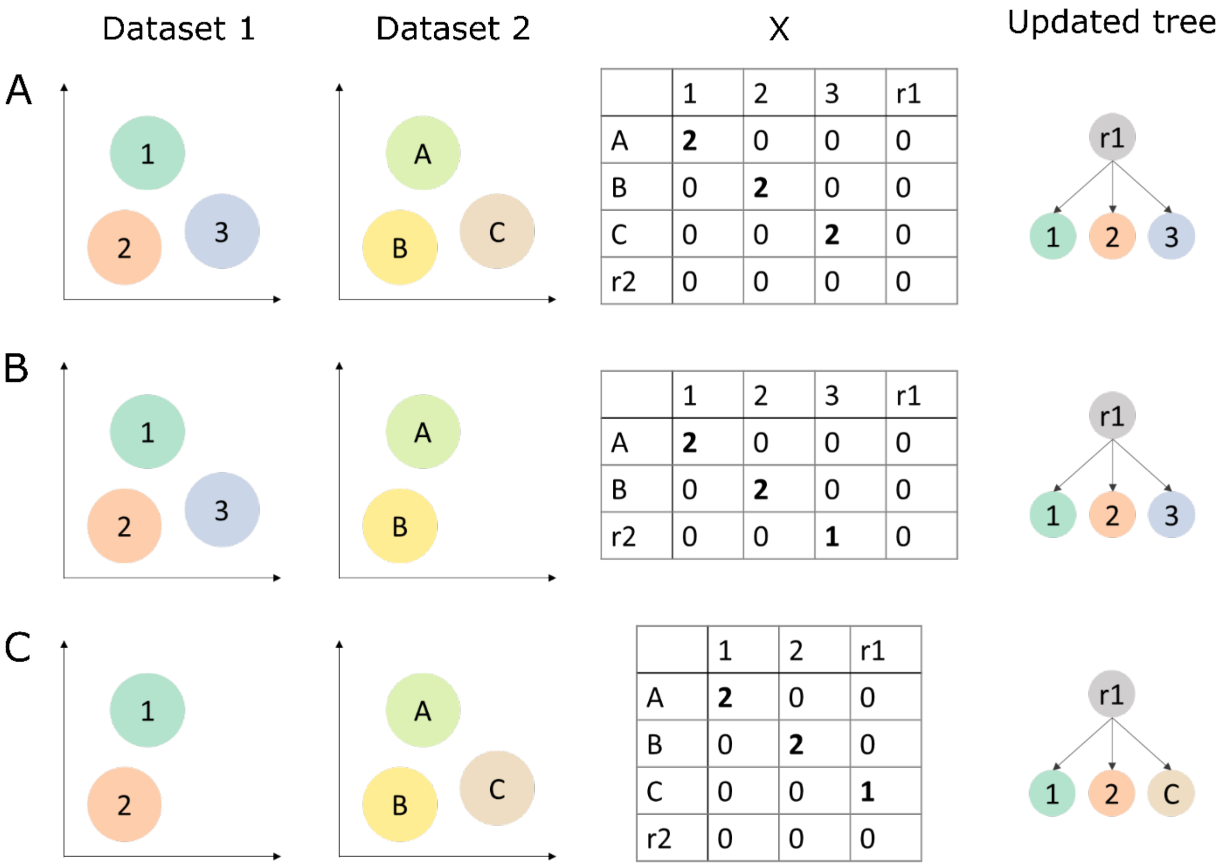

A

	1	2	3	r1
A	**2**	0	0	0
B	0	**2**	0	0
C	0	0	**2**	0
r2	0	0	0	0

B

	1	2	3	r1
A	**2**	0	0	0
B	0	**2**	0	0
r2	0	0	**1**	0

C

	1	2	r1
A	**2**	0	0
B	0	**2**	0
C	0	0	**1**
r2	0	0	0

Supplementary Fig. 24 Schematic examples of the simple scenarios. For each scenario, we show what the cell populations in the two datasets could look like, X and the updated tree.

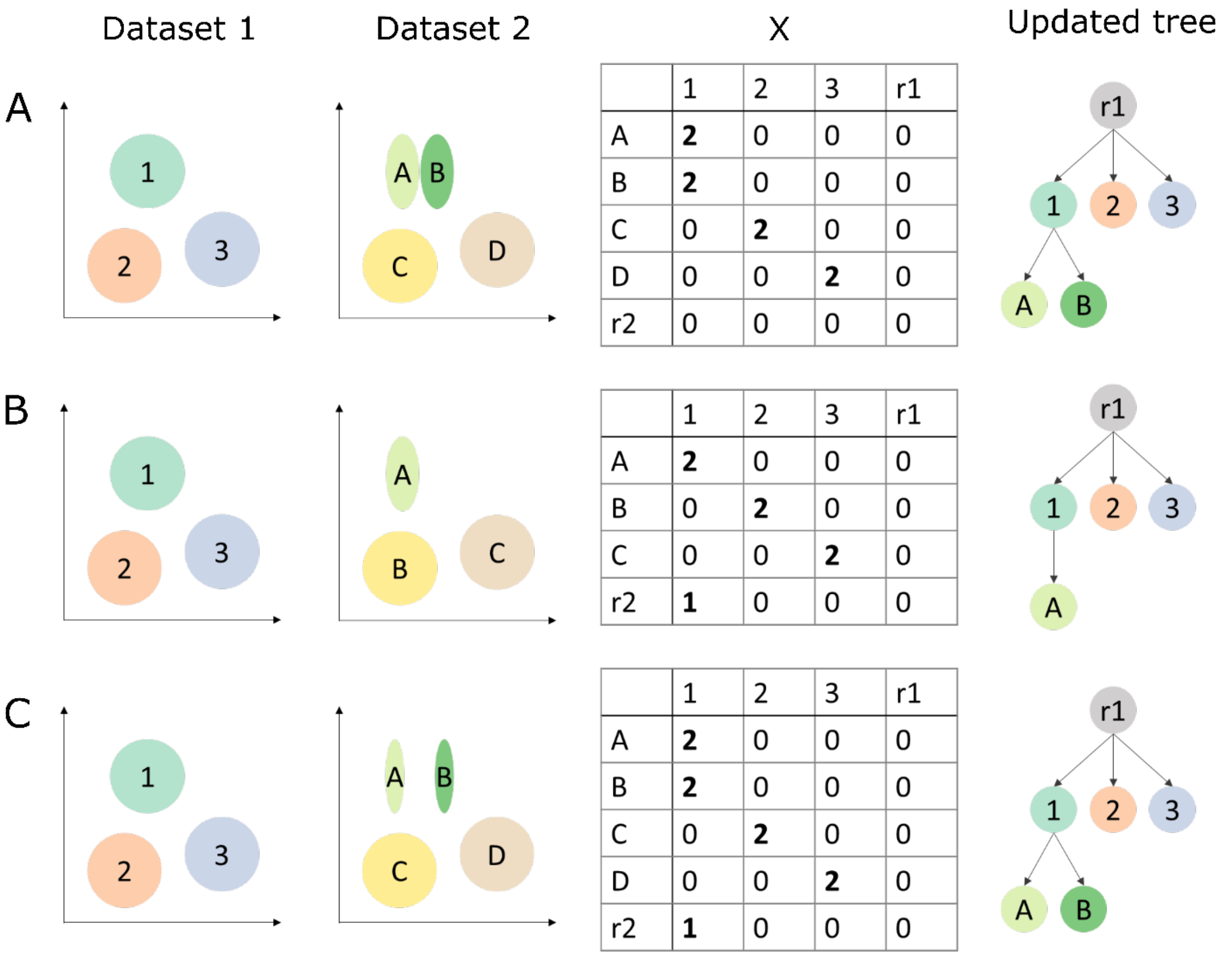

A

	1	2	3	r1
A	**2**	0	0	0
B	**2**	0	0	0
C	0	**2**	0	0
D	0	0	**2**	0
r2	0	0	0	0

B

	1	2	3	r1
A	**2**	0	0	0
B	0	**2**	0	0
C	0	0	**2**	0
r2	**1**	0	0	0

C

	1	2	3	r1
A	**2**	0	0	0
B	**2**	0	0	0
C	0	**2**	0	0
D	0	0	**2**	0
r2	**1**	0	0	0

Supplementary Fig. 25 Schematic examples of the colsums scenarios. For each scenario, we show what the cell populations in the two datasets could look like, X and the updated tree.

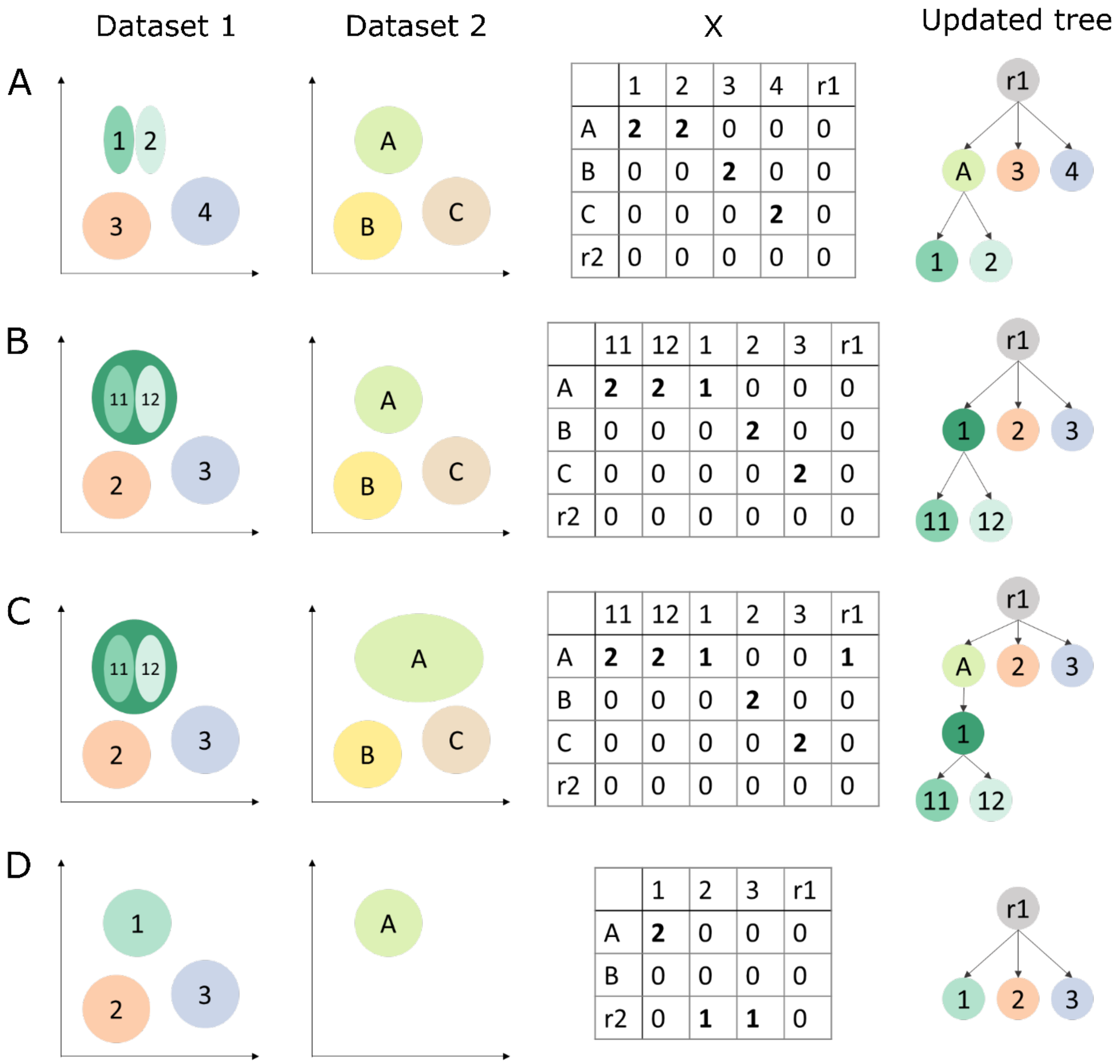

Supplementary Fig. 26 Schematic examples of the rowsums scenarios. For each scenario, we show what the cell populations in the two datasets could look like, X and the updated tree.

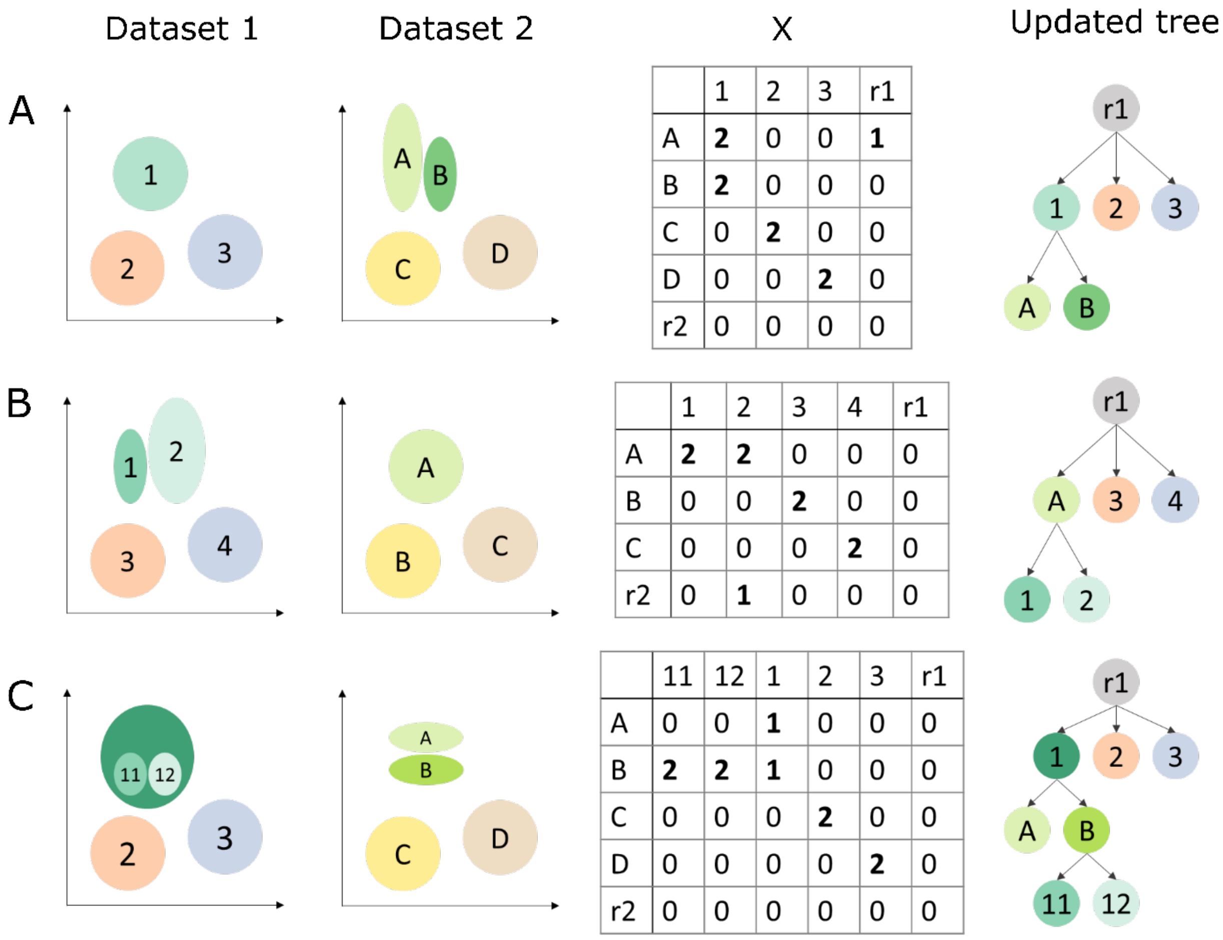

A

	1	2	3	r1
A	**2**	0	0	**1**
B	**2**	0	0	0
C	0	**2**	0	0
D	0	0	**2**	0
r2	0	0	0	0

B

	1	2	3	4	r1
A	**2**	**2**	0	0	0
B	0	0	**2**	0	0
C	0	0	0	**2**	0
r2	0	**1**	0	0	0

C

	11	12	1	2	3	r1
A	0	0	**1**	0	0	0
B	**2**	**2**	**1**	0	0	0
C	0	0	0	**2**	0	0
D	0	0	0	0	**2**	0
r2	0	0	0	0	0	0

Supplementary Fig. 27 Schematic examples of the complex scenarios. For each scenario, we show what the cell populations in the two datasets could look like, X and the updated tree.

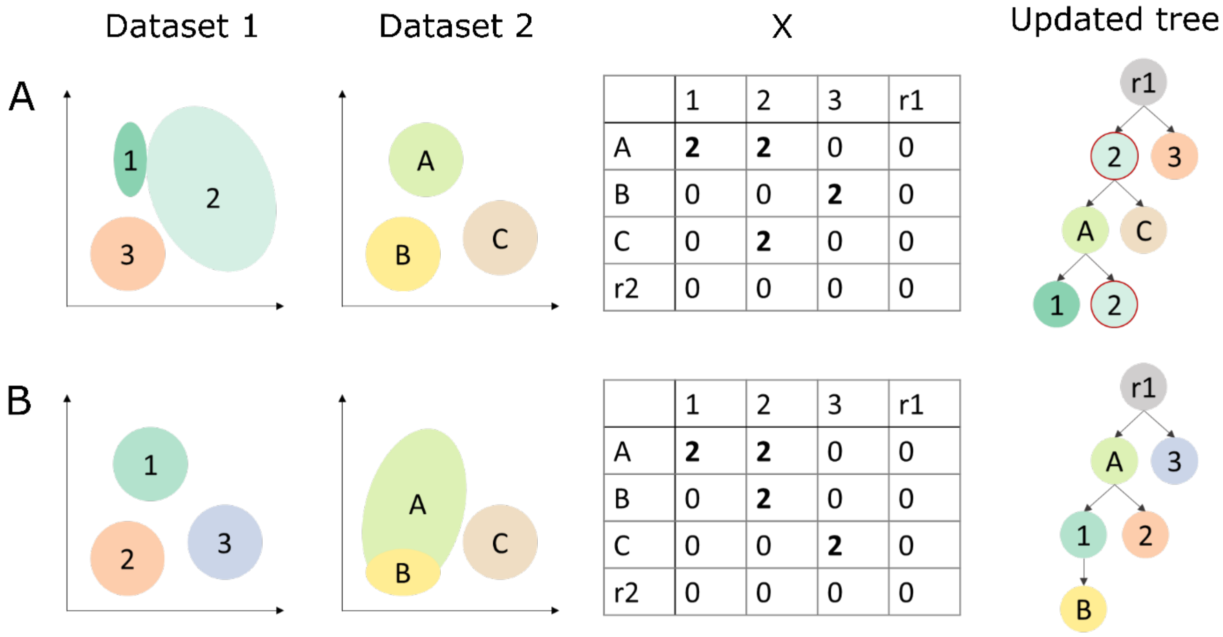

A

	1	2	3	r1
A	**2**	**2**	0	0
B	0	0	**2**	0
C	0	**2**	0	0
r2	0	0	0	0

B

	1	2	3	r1
A	**2**	**2**	0	0
B	0	**2**	0	0
C	0	0	**2**	0
r2	0	0	0	0

Supplementary Fig. 28 Schematic examples of the impossible scenarios. For each scenario, we show what the cell populations in the two datasets could look like, X and why the updated tree is not possible.

Supplementary tables

Supplementary Table 1 Explanation of the most common matching scenarios.

Scenario	Biological explanation	Identification in the matching matrix (*X*)	Solution
Perfect match	Population *i* of dataset 1 exactly matches population *j* of dataset 2	$X_{j,i} = 1$ or *2* rest of row *j* and column *i* is zero	Rename labels of population *j* to population *i*
Splitting populations	Multiple populations from dataset 2 are subpopulations of population *i* of dataset 1	Multiple non-zero values in column *i* of *X*	Add corresponding populations from dataset 2 as children to population *i*
Merging populations	Multiple populations from dataset 1 are subpopulations of population *j* of dataset 2	Multiple non-zero values in column *j* of *X*	Add population *j* as a parent to the corresponding populations of dataset 1
New population	Dataset 2 contains a new, unseen population *j*	$X_{j,root1} = 1$, the rest of row *j* is zero	Add population *j* as a child to the root node.

Supplementary Table 2 Labels of the simulated dataset when testing tree construction

Original label	Label Batch 1	Label Batch 2	Label Batch 3
Group1	Group12	Group1	Group1
Group2	Group12	Group2	Group2
Group3	Group3	Group3	Group3
Group4	Group456	Group4	Group4
Group5	Group456	Group56	Group5
Group6	Group456	Group56	Group6

Supplementary Table 3 Labels of PBMC-FACS dataset when testing tree construction

Original label	Label Batch 1	Label Batch 2	Label Batch 3
CD14+ Monocytes	CD14+ Monocytes	CD14+ Monocytes	CD14+ Monocytes
CD19+ B-cells	CD19+ B-cells	CD19+ B-cells	CD19+ B-cells
CD56+ NK cells	CD56+ NK cells	CD56+ NK cells	CD56+ NK cells
CD4+ T-cells	T-cells	CD4+ T-cells	-
CD4+/CD25+ reg. T-cells	T-cells	-	CD4+/CD25+ reg. T-cells
CD4+/CD45RA+/CD25-naive T-cells	T-cells	-	CD4+/CD45RA+/CD25-naive T-cells
CD4+/CD45RO+ mem. T-cells	T-cells	-	CD4+/CD45RO+ mem. T-cells
CD8+ T-cells	T-cells	CD8+ T-cells	-
CD8+/CD45RA+ naïve T-cells	T-cells	-	CD8+/CD45RA+ naïve T-cells

Supplementary Table 4 Confusion matrix of the linear SVM on the PBMC data. Here, the linear SVM was trained using the predefined hematopoietic tree.

	CD14+ MC	CD19+ B	CD34+	CD56+ NK	CD4+ T	CD4+ T Reg	CD4+ T Naive	CD4+ T Memory	CD8+ T	CD8+ T Naive	root
CD14+ MC	1957	1	0	1	2	15	0	1	0	0	23
CD19+ B	0	1998	0	0	0	0	0	1	0	0	1
CD34+	1	26	1794	1	0	0	0	0	6	0	172
CD56+ NK	0	0	2	1988	0	0	0	1	6	0	3
CD4+ T	1	0	0	0	191	1010	790	3	0	2	3
CD4+ T Reg	0	0	0	0	113	1704	175	4	0	0	4
CD4+ T Naive	1	1	0	0	94	140	1751	1	1	9	2
CD4+ T Memory	0	0	3	0	1	33	14	1888	32	26	3
CD8+ T	0	0	0	0	1	5	0	44	1884	65	1
CD8+ T Naive	0	1	0	0	3	0	13	36	31	1916	0

Supplementary Table 5 Confusion matrix of the linear SVM on the PBMC data. Here, the linear SVM was trained on the learned tree. The CD4+ memory T-cells are a subpopulation of CD8+ T-cells now.

	CD14+ MC	CD19+ B	CD34+	CD56+ NK	CD4+ T	CD4+ T Reg	CD4+ T Naive	CD4+ T Memory	CD8+ T	CD8+ T Naive	root
CD14+ MC	1957	1	0	1	0	17	0	1	0	0	23
CD19+ B	0	1998	0	0	0	0	0	0	0	1	1
CD34+	1	26	1794	1	0	0	0	2	0	4	172
CD56+ NK	0	0	2	1988	0	0	0	7	0	0	3
CD4+ T	1	0	0	0	0	1116	875	5	0	0	3
CD4+ T Reg	0	0	0	0	0	1794	200	2	0	0	4
CD4+ T Naive	1	1	0	0	0	133	1860	2	0	1	2
CD4+ T Memory	0	0	3	0	0	4	0	1972	0	18	3
CD8+ T	0	0	0	0	0	2	0	774	0	1223	1
CD8+ T Naive	0	1	0	0	0	0	3	24	0	1972	0

Supplementary Table 6 Labels of the simulated dataset when testing tree construction with missing cell populations

Original label	Label Batch 1	Label Batch 2	Label Batch 3
Group1	Group12	Group1	Group1
Group2	Group12	Group2	Group2
Group3	Group3	Group3	Group3
Group4	Group456	Group4	Group4
Group5	Group456	-	Group5
Group6	Group456	Group6	Group6

Supplementary Table 7 Characteristics of the brain datasets

	Year	Protocol	Number of cells	Number of cell populations
**Tabula Muris**	2018	Smart-seq2	7,856	9
**Rosenberg**	2018	SPLiT-seq	76,322	73
**Zeisel**	2018	10Xv1	85,621	11, 30
**Saunders**	2018	Drop-seq	389,439	11, 437
